# Histopathological biomarkers for predicting the tumour accumulation of nanomedicines

**DOI:** 10.1038/s41551-024-01197-4

**Published:** 2024-04-08

**Authors:** Jan-Niklas May, Jennifer I. Moss, Florian Mueller, Susanne K. Golombek, Ilaria Biancacci, Larissa Rizzo, Asmaa Said Elshafei, Felix Gremse, Robert Pola, Michal Pechar, Tomáš Etrych, Svea Becker, Christian Trautwein, Roman D. Bülow, Peter Boor, Ruth Knuechel, Saskia von Stillfried, Gert Storm, Sanyogitta Puri, Simon T. Barry, Volkmar Schulz, Fabian Kiessling, Marianne B. Ashford, Twan Lammers

**Affiliations:** 1 Institute for Experimental Molecular Imaging, University Hospital RWTH Aachen, Aachen, Germany; 2 Early TDE Discovery, Oncology R&D, AstraZeneca, Cambridge, UK; 3 Gremse-IT GmbH, Aachen, Germany; 4 Institute of Macromolecular Chemistry, Czech Academy of Sciences, Prague, Czech Republic; 5 Clinic for Gastroenterology, Metabolic Disorders, and Internal Intensive Medicine, University Hospital RWTH Aachen, Aachen, Germany; 6 Center for Integrated Oncology Aachen Bonn Cologne Duesseldorf, Aachen, Germany; 7 Institute of Pathology, University Hospital RWTH Aachen, Aachen, Germany; 8 Department of Pharmaceutics, Utrecht University, Utrecht, the Netherlands; 9 Department of Biomaterials, Science and Technology, University of Twente, Enschede, the Netherlands; 10 Department of Surgery, Yong Loo Lin School of Medicine, National University of Singapore, Singapore, Singapore; 11 Advanced Drug Delivery, Pharmaceutical Sciences, R&D, AstraZeneca, Macclesfield, UK; 12 Fraunhofer Institute for Digital Medicine MEVIS, Aachen, Germany; 13 Physics Institute III B, RWTH Aachen University, Aachen, Germany

**Keywords:** Predictive markers, Tumour biomarkers, Drug delivery

## Abstract

The clinical prospects of cancer nanomedicines depend on effective patient stratification. Here we report the identification of predictive biomarkers of the accumulation of nanomedicines in tumour tissue. By using supervised machine learning on data of the accumulation of nanomedicines in tumour models in mice, we identified the densities of blood vessels and of tumour-associated macrophages as key predictive features. On the basis of these two features, we derived a biomarker score correlating with the concentration of liposomal doxorubicin in tumours and validated it in three syngeneic tumour models in immunocompetent mice and in four cell-line-derived and six patient-derived tumour xenografts in mice. The score effectively discriminated tumours according to the accumulation of nanomedicines (high versus low), with an area under the receiver operating characteristic curve of 0.91. Histopathological assessment of 30 tumour specimens from patients and of 28 corresponding primary tumour biopsies confirmed the score’s effectiveness in predicting the tumour accumulation of liposomal doxorubicin. Biomarkers of the tumour accumulation of nanomedicines may aid the stratification of patients in clinical trials of cancer nanomedicines.

## Abstract (web summary)

The densities of blood vessels and of tumour-associated macrophages are key predictive features of the degree of accumulation of polymeric and liposomal nanomedicines, as shown for specimens of mouse and human tumours.

## Main

Nanomedicines hold potential for improving cancer therapy^1–3^. Their clinical translation, however, has not met expectations, partly because of a lack of biomarkers for patient stratification^4,5^. In drug development for oncology, biomarkers and companion diagnostics are extensively employed for patient stratification, typically from advanced preclinical stages onwards. Biomarkers and patient-stratification protocols help to address the high heterogeneity that is typical of cancer, and they have been crucial in ensuring the clinical development of molecularly targeted drugs, such as kinase inhibitors, therapeutic antibodies and antibody–drug conjugates^6,7^.

Remarkably, no biomarkers have been established yet for capturing the tumour-targeted drug-delivery process and for guiding patient stratification in clinical trials for cancer nanomedicine. Considering that tumour accumulation is crucial for good therapeutic outcome and, conversely, that individuals not showing good tumour accumulation should be excluded from clinical trials of cancer nanomedicines, it is imperative to establish probes or protocols for quantitative assessment and accurate prediction of nanomedicine target-site localization. It has already been shown in mouse models and patients with cancer that the tumour accumulation of companion diagnostics^8–11^ and nanomedicine theranostics^12–14^ corresponds well with treatment outcomes from nanomedicines, particularly if patients are not pre-treated too heavily before being enroled in clinical trials. While in principle highly quantitative and properly predictive, monitoring nanomedicine tumour targeting via magnetic resonance imaging, computed tomography (CT) and positron emission tomography (PET) is not straightforward and not very time efficient and cost efficient^4^. Non-invasive imaging furthermore requires access to specialized facilities, including radiochemistry labs and advanced instrumentation, which are not widely available in community hospitals. These notions complicate the use of non-invasive imaging for patient stratification in (nano)drug development, clinical translation and routine practice.

In this Article, to establish a more pragmatic alternative for non-invasive imaging, we set out to explore the use of histopathological biomarkers in tumour tissue for enabling patient stratification in clinical trials of cancer nanomedicines. Tumour biopsies are readily available for almost all patients with cancer, and they are routinely used for disease diagnosis, staging and therapy selection. We reason that microenvironmental features ingrained in the pathophysiological makeup of tumours, such as their vascularization and stroma composition, are potentially key enablers of tumour-directed drug delivery. Accordingly, we hypothesized that the histopathological assessment of biomarkers in tumour tissue may serve as a pragmatic way forward towards patient stratification.

## Results

### Quantification of the accumulation of nanomedicine in tumours

**Fig. 1:**
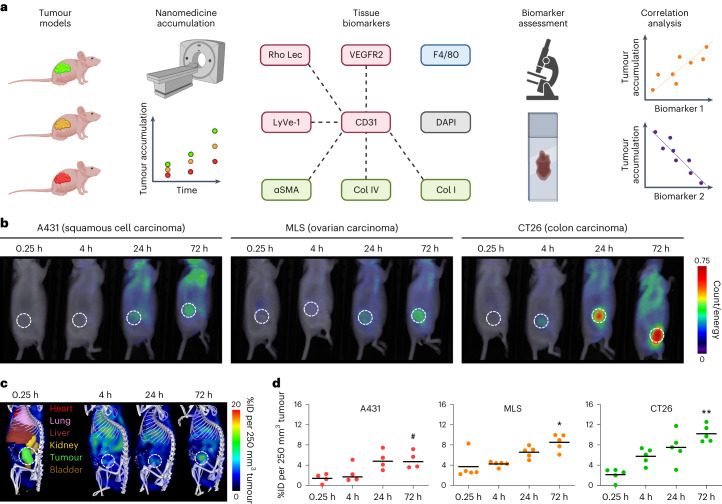
Towards prediction of nanomedicine tumour targeting via tissue biomarkers. **a**, A schematic of the experimental protocol aimed at identifying tumour-tissue biomarkers that correlate with nanomedicine accumulation in tumours. The tumour accumulation of the prototypic polymeric nanocarrier, PHPMA, was assessed using CT–FMT in three distinct mouse models with varying degrees of tumour targeting. Subsequently, correlation analyses were conducted using 23 tumour-tissue microenvironment features associated with tumour-targeted drug delivery, focusing on aspects related to the vasculature (red), stroma (green), macrophages (blue) and cellular density (grey). The dashed lines indicate double stained features. For further details, please refer to Supplementary Table 1. The illustration was created with BioRender.com. **b**, FRI-based, longitudinal optical imaging of DY750-labelled PHPMA accumulation in the tumours of mice with A431, MLS and CT26 tumours representing low, medium and high levels of target-site accumulation, respectively (the white dashed circles indicate tumour location, and one mouse per tumour model is shown). **c**,**d**, Longitudinal CT–FMT visualization (**c**) and quantification of DY750-labelled PHPMA tumour accumulation (**d**) in percent of the injected dose (100% is equal to 2 nmol of dye) normalized to 250 mm³ tumour volume. The statistical significance between the two models was assessed via individual Student’s *t*-tests (A431 versus MLS, *P = 0.0168; A431 versus CT26, **P = 0.0025) and between all models via one-way ANOVA (^#^*P* *=* 0.0024). Each data point represents a CT–FMT scan of one animal.

We first determined nanomedicine tumour accumulation in three mouse models with differing degrees of vascularization, stroma composition and target-site localization (Fig. 1a). The tumour models were A431 human epidermoid carcinoma, MLS human ovarian carcinoma and CT26 murine colon cancer. As a nanocarrier, we employed a 67 kDa-sized poly(*N*-(2-hydroxypropyl) methacrylamide) (PHPMA) polymer, as this prototypic albumin-sized macromolecule has consistently provided us with high levels of tumour accumulation in a variety of models^15–17^. We used fluorescence reflectance imaging (FRI) and hybrid CT–FMT to visualize and quantify the biodistribution and tumour accumulation of DY750-labelled PHPMA (Fig. 1b,c and Supplementary Fig. 1). When normalized to average tumour volume at the timepoint of analysis (250 mm^3^), at 72 h post intravenous (i.v.) injection, we found average levels of target-site localization of 5.0 ± 1.7, 8.5 ± 1.6 and 10.2 ± 1.7 percent of the injected dose (%ID) for A431, MLS and CT26 tumours, respectively, exemplifying sustained localization to tumours over time, as well as different accumulation patterns in the three models (*P* = 0.0024, one-way analysis of variance (ANOVA); Fig. 1d and Supplementary Fig. 1). The tumours were then excised, and DY750-labelled PHPMA accumulation patterns were validated ex vivo using FRI (Supplementary Fig. 2). The collected tumours were fixed, sectioned and stained for biomarker assessment.

### Analysis of tumour-tissue biomarkers

We analysed 23 tumour microenvironment features associated with tumour-targeted drug delivery (Supplementary Table 1). These included vascular features, such as vessel density (CD31), perfusion (lectin) and angiogenesis (VEGFR2); lymph vessels (LYVE-1); extracellular matrix components, such as αSMA, collagen I and collagen IV; tumour-associated macrophages (TAM; F4/80); and tumour cell density (4,6-diamidino-2-phenylindole). In addition, we analysed combinations of the above, via immunofluorescent double-stainings, to, for example, assess vessel support (αSMA^+^/CD31^+^), vessel function (lectin^+^/CD31^+^) and the fraction of angiogenic vessels (VEGFR2^+^/CD31^+^).

**Fig. 2:**
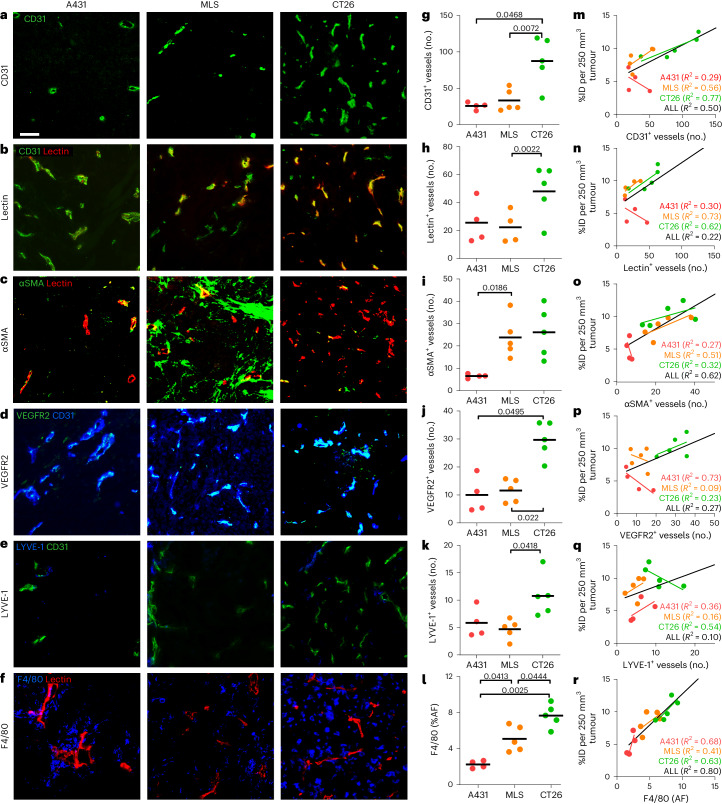
Histopathological biomarker assessment and correlation with nanomedicine tumour targeting. **a**–**f**, Immunofluorescence stainings for all blood vessels (CD31) (**a**), actively perfused vessels (lectin) (**b**), pericyte-supported vessels (αSMA) (**c**), angiogenic vessels (VEGFR2) (**d**), lymphatic vessels (LYVE-1) (**e**) and TAM (F4/80) (**f**) in A431, MLS and CT26 tumours. Scale bar, 50 µm. **g**–**l**, Quantification of the immunofluorescence images for CD31^+^ vessels (**g**), lectin^+^ vessels (**h**), αSMA^+^ vessels (**i**), VEGFR2^+^ vessels (**j**), LYVE-1^+^ vessels (**k**) and F4/80 (**l**) (no., number). The black bars indicate means. *P < 0.05, **P < 0.01 (Student’s *t*-test). Note that the analysis in **g**–**i** is based on 10× magnification images, while the analysis in **j**–**l** is based on 20× magnification. **m**–**r**, Correlation of PHPMA tumour accumulation at 72 h post injection (in percent of the injected dose (100% represents 2 nmol of dye) normalized to 250 mm³ tumour volume) with the respective tumour-tissue biomarker features (CD31^+^ vessels (**m**), lectin^+^ vessels (**n**), αSMA^+^ vessels (**o**), VEGFR2^+^ vessels (**p**), LYVE-1^+^ vessels (**q**) and F4/80 (**r**)). The trendlines are shown per tumour model (colour-coded) and for all tumours together (black). The *R*^2^ values indicate the coefficient of determination and reflect the goodness of fit. Each data point represents one animal.

The tumour-tissue biomarkers were captured and quantified via fluorescence microscopy and correlated with nanocarrier accumulation in A431, MLS and CT26 tumours (Fig. 2). Regarding blood vessel density and perfusion, we observed an overall good agreement between the number of (perfused) vessels and DY750-labelled PHPMA accumulation. The CT26 tumours had the highest number of total and functional blood vessels (89.0 ± 35.9 and 48.0 ± 18.8, respectively; Fig. 2a,b,g,h), and this was in line with their high level of polymer accumulation (10.2 ± 1.7 %ID per 250 mm^3^; Fig. 1d). Conversely, A431 tumours had low levels of total and functional blood vessels (28.5 ± 15.1 and 25.6 ± 15.5, repectively; Fig. 2a,b,g,h), aligning with their low accumulation of DY750-labelled PHPMA (5.0 ± 1.7 %ID per 250 mm^3^; Fig. 1d). Interestingly, while CT26 tumours had the highest absolute numbers of total and functional blood vessels, A431 tumours presented with the highest relative level of perfused vessels (91.3%, as compared with 62.7% for MLS and 54.9% for CT26; Supplementary Fig. 3j). This indicates that the absolute number of (functional) blood vessels is a more important factor determining nanomedicine tumour targeting than the relative fraction of vascular perfusion. In good agreement with this, also the absolute numbers of αSMA^+^, Col I^+^, Col IV^+^ and VEGFR2^+^ blood vessels (Fig. 2c,d,i,j) correlated better with DY750-labelled PHPMA tumour accumulation than the relative fractions of αSMA^+^, Col I^+^, Col IV^+^ and VEGFR2^+^ vessels (Supplementary Fig. 3j–n).

Regarding the retention component of nanomedicine tumour targeting, we particularly looked at LYVE-1^+^ lymphatic vessels and F4/80^+^ TAM. Interestingly, we observed that the tumour model with the highest level of PHPMA accumulation, that is, CT26, had almost double the number of LYVE-1^+^ lymphatic vessels as A431 and MLS (Fig. 2e,k). This indicates that the absence of effective lymphatics as a mediator of nanomedicine retention in tumours may be less important than originally anticipated^18^. It actually even suggests the opposite, which is that a certain degree of functional lymphatics in tumours may be needed to assist in attenuating the high interstitial fluid pressure that is typical of tumours^19^. A very good correlation was found between the density of TAM and nanomedicine accumulation (Fig. 2f,l,r). The area fraction of TAM increased from 2.2% to 5.1% to 7.7% for A431, MLS and CT26 tumours, respectively, correlating almost linearly with the increased tumour accumulation in these models (Fig. 1d) and resulting in good *R*^2^ values both within and across the three models (Fig. 2r). This finding corroborates an increasing number of notions that TAM act as a key reservoir for nanomedicine retention in tumours^8,20^. It furthermore implies that TAM density seems to be a suitable tumour-tissue biomarker to predict nanomedicine tumour accumulation.

### Machine learning identifies tumour-tissue biomarkers and feature importance

**Fig. 3:**
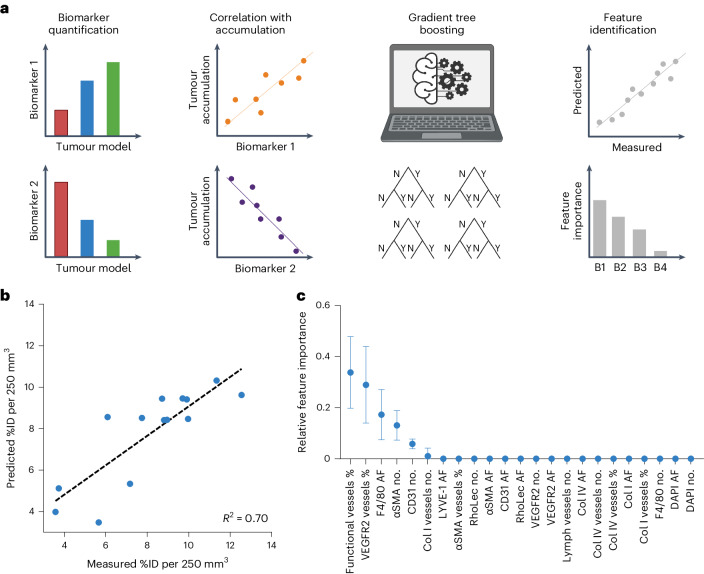
Identifying the importance of histopathological biomarker features using GTB. **a**, Schematic workflow. Tumour-tissue biomarkers were stained, quantified and correlated with the tumour accumulation of PHPMA nanocarriers. GTB-based machine learning was employed to rank feature importance using predicted versus measured PHPMA tumour accumulation values (Y, yes; N, no; B1–4, biomarker 1–4). **b**, *N*-fold cross-validation of predicted versus measured PHPMA tumour accumulation patterns illustrates the accuracy of the employed GTB method for predicting nanomedicine tumour targeting (in percent of the injected dose (100% represents 2 nmol of dye) normalized to 250 mm³ tumour volume). **c**, Ranking of the importance of the identified tumour-tissue biomarker features based on their assignment in the GTB decision trees (%, biomarker positive vessels of the number total vessels; no., number). The error bars indicate the standard deviaitoin (*n* = 14).

Feature importance was assessed using gradient tree boosting (GTB). GTB is a machine learning technique for building predictive regression models based on a set of yes/no decision trees^21–23^. The trained GTB model considered all 23 features analysed as a regression model and was applied to predict polymeric nanomedicine tumour accumulation (Fig. 3a). Given the relatively small dataset, the leave-one-out method was employed to avoid the mixing of training and testing datasets. Ten decision trees, with a depth of up to eight questions, were found to be able to properly predict nanocarrier tumour accumulation based on histopathological features (*R*² = 0.70; Fig. 3b). As exemplified in Fig. 3c, GTB-based importance assessment identified the percentage of lectin^+^ (that is, functional vessels percentage) and angiogenic (that is, VEGFR2 vessels percentage) blood vessels, the density of TAM (that is, F4/80 area fraction (AF)) and the total, αSMA^+^ and Col I^+^ number of blood vessels (that is, CD31 number, αSMA number and Col I vessels number, respectively) as predictive features.

When aiming to establish a biomarker for patient stratification, the practicality of the approach and the presence of a proper dynamic range are crucial. This implies that in the features identified via GTB, the functionality of tumour blood vessels needs to be excluded, because lectin cannot be injected in patients. For the fraction of VEGFR2^+^ blood vessels, the dynamic range is small (Supplementary Fig. 3l), making it unlikely to serve as a good biomarker. Moreover, as for the number of αSMA^+^ and Col1^+^ blood vessels, double-staining would be required. This can be done preclinically with immunofluorescence, but is not typically performed in histopathological protocols in routine clinical practice. In follow-up studies with additional tumour models, we therefore focused on blood vessel and TAM density as tissue biomarkers.

### Validation of tumour-tissue biomarkers in ten patient-derived (PDX) and cell-line-derived (CDX) models

**Fig. 4:**
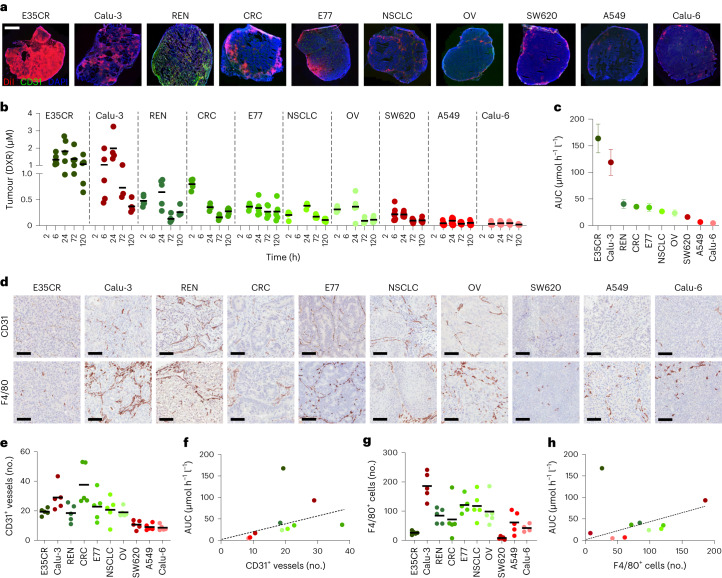
Liposome accumulation and biomarker correlation in ten (six PDX and four CDX) tumour models. **a**, Fluorescence microscopy analysis of Dil-labelled PEGylated liposomes (in red) in ten tumour models at 24 h after i.v. administration Scale bar, 200 µm. The blood vessels are stained in green and the cell nuclei in blue. **b**, Tumour accumulation of PEGylated liposomal DXR in six PDX (green dots) and four CDX (red dots) tumour models. Individual and mean (black bars) tumour concentrations of DXR are shown for 20 mice per group and 5 mice per timepoint. **c**, Total tumour accumulation over time of PEGylated liposomal DXR (that is, AUC_0–120h_). Values represent mean ± standard error of the mean. **d**, Histopathological DAB staining of tumour blood vessels (CD31) and TAM (F4/80) for the ten models. Scale bars, 100 µm. **e**–**h**, Quantification of blood vessel (**e**) and TAM (**g**) density based on DAB staining and correlation of blood vessel (**f**) and TAM (**h**) density with total liposomal DXR tumour accumulation (no., number of vessels or TAM per field of view).

The feature importance and biomarker potential of tumour blood vessels and TAM were confirmed in a panel of ten tumour models. This panel was selected to encompass models with very different tumour microenvironment architectures (thereby reflecting the heterogeneity observed in human tumours^24^) and consisted of six PDX and four CDX xenograft models. To ensure broad applicability of blood vessel and TAM density as biomarkers for predicting nanomedicine accumulation, we decided to employ a second drug-delivery system in these ten models, replacing the prototypic polymeric nanocarrier PHPMA with a PEGylated liposome formulation similar to Doxil/Caelyx^25^. Initially, fluorescent DiI-labelled liposomes were used to visualize the accumulation and distribution of liposomes in tumours. The highest levels of liposome accumulation were observed in E35CR and Calu-3 tumours, and the lowest levels were found in A549 and Calu-6 tumours (Fig. 4a).

We next used doxorubicin (DXR)-loaded liposomes and determined drug accumulation in tumours using high-performance liquid chromatography. For each of the ten models, this was done for four timepoints, with five tumours per timepoint (Fig. 4b). Total DXR concentrations over time were quantified and expressed as the area under the curve (AUC). In good agreement with the DiI-liposome fluorescence data (Fig. 4a), AUC determination demonstrated that tumour DXR concentrations were highest in E35CR and Calu-3, making these the highest drug-accumulating models, with drug levels three to five times higher than those of the majority of other models (Fig. 4c). A549 and Calu-6 were again found to accumulate the lowest amounts of liposomes, with DXR concentrations five to ten times lower than most other models. Interestingly, when comparing all AUC values together, it was furthermore found that PDX models presented with higher overall levels of liposomal DXR accumulation than CDX models (Fig. 4c).

In clinical practice, pathology protocols involve light (and not fluorescence) microscopy. Accordingly, we switched to 3,3’-diaminobenzidine (DAB) staining and studied blood vessel and TAM density via standard histopathology in the ten PDX and CDX models. As shown in Fig. 4d–h, we found that the three models with the lowest accumulation levels upon administration of liposomal DXR, that is, SW620, A549 and Calu-6 models (Fig. 4c), also presented with the lowest levels of CD31 and F4/80 staining. Across the ten different tumour models, there was a good correlation between tumour blood vessel and TAM density and nanomedicine accumulation (Fig. 4f,h). It should be noted in this regard, however, that the E35CR model was identified as a clear outlier, as it presented with the highest levels of Dil- and DXR-loaded liposome accumulation (Fig. 4a–c), while its levels of CD31^+^ blood vessels were intermediate (Fig. 4f) and those of F4/80^+^ TAM were very low (Fig. 4g). When determining the area fraction of CD31 and F4/80 instead of the number of CD31^+^ and F4/80^+^ cells, observations were identical for all of the above notions, confirming the robustness of the tumour-tissue biomarkers identified (Supplementary Fig. 4). Altogether, these results demonstrate that there is a good correlation between the levels of the tumour blood vessels and TAM and the level of nanomedicine tumour accumulation.

### Blood vessel and TAM product score predicts nanomedicine tumour targeting

**Fig. 5:**
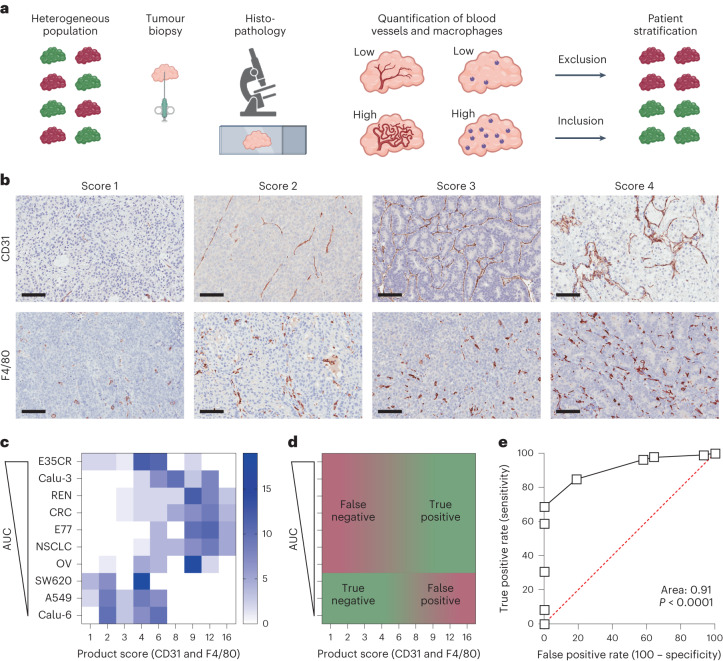
Histopathological biomarker product score for predicting nanomedicine tumour targeting. **a**, Schematic workflow demonstrating the concept of patient stratification in cancer nanomedicine clinical translation based on tumour-tissue biopsies, created with BioRender.com. **b**, DAB staining illustrating the density of tumour blood vessels (CD31) and TAM (F4/80) in tumours, reaching from lowest (score 1) to highest (score 4) levels of blood vessel and macrophage density. Biomarker scores indicate 1 for absent, 2 for low, 3 for intermediate and 4 for high. Scale bars, 100 µm. **c**, Colour-coded heatmap, representing the distribution of CD31 and F4/80 product scores in the ten PDX and CDX tumour models with differing degrees of PEGylated liposomal DXR tumour accumulation. Tumours are ranked from high to low AUC, from top to bottom. Tumour-tissue biomarkers were scored by ten blinded observers, who each analysed three tissue sections per tumour model (*n* = 30 in total). The colour intensity reflects the number of product scores. **d**, Schematic displaying the distribution of true and false positives and negatives in the tumour-tissue biomarker product score heatmap. **e**, Receiver operating characteristic (ROC) curve, generated on the basis of the tumour-tissue biomarker product scores, exemplifying very high diagnostic accuracy differentiating between low and high nanomedicine tumour accumulation (ROC curve is based on the scores in **c**; the red dashed line represents randomness and the units of the axis are in %).

Having identified tumour blood vessels and TAM as key features correlating with nanomedicine tumour accumulation, we next explored the robustness, validity and potential clinical applicability of combined tumour blood vessel and macrophage scoring, with the aim of developing a simple and straightforward biomarker protocol for patient stratification. This protocol is primarily designed to help predict which individuals from a heterogeneous patient population should be excluded in clinical trials, because their tumours are likely to show low nanomedicine accumulation and poor therapeutic efficacy (Fig. 5a).

We conceived a DAB-based histopathological scoring setup in which we considered 1 for absent, 2 for low, 3 for intermediate and 4 for high for the expression of both tumour-tissue biomarkers (Fig. 5b). Ten blinded observers, including three board-certified pathologists, were asked to score 60 tumour sections (30 for CD31 and 30 for F4/80; 6 for each tumour model). As shown in Fig. 5c, the colour-coded scoring intensities demonstrate that for tumour models with low CD31 and F4/80 product scores, the levels of liposomal DXR accumulation were also low. With a cut-off score of 6 to differentiate between tumours with low versus high nanomedicine accumulation, the blinded observers’ product scores correctly identified SW620, A549 and Calu-6 as true negatives (Figs. 4a–c and 5c,d). Conversely, six out of seven models with good nanomedicine accumulation were correctly identified as true positives (Fig. 5c, d). The E35CR model turned out to be false negative, as its low CD31 and F4/80 product score incorrectly indicated that it would not accumulate liposomes well, which it clearly did do (Fig. 4a–c). No false positives were detected (Fig. 5c,d). Altogether, nine out of ten tumour models could be correctly associated with low versus high nanomedicine accumulation on the basis of our tumour blood vessel and TAM biomarker product score.

To quantify the biomarker performance of our product score, we determined the area under the receiver operating characteristics (AUROC) curve. The AUROC curve represents a probability assessment, with a value of 0.5 resulting in a straight 45°-line reflecting randomness (represented by the dashed red line in Fig. 5e). The AUROC curve represents the capability of a biomarker to distinguish between different classes, in this case between low versus high nanomedicine tumour accumulation. We obtained an AUROC value of 0.91 for our blood vessel and TAM product score (Fig. 5e), which is generally considered excellent for predicting nanomedicine tumour targeting, following the published criteria^26^.

### Validation of biomarker product score in immunocompetent mice and in patient datasets

The robustness and translatability of our biomarker product score were assessed in immunocompetent mouse models and in patient samples. The former were included to rule out the possibility that the presence of T cells plays an important role in determining nanomedicine delivery to tumours. To this end, we analysed PHPMA accumulation in orthotopic 4T1 triple-negative breast cancer tumours in BALB/c mice and PEGylated liposome accumulation in subcutaneous and orthotopic Hep55.1C liver tumours in C57BL/6J mice. As shown in Supplementary Fig. 5, good correlations between blood vessel and TAM product scores and nanomedicine tumour targeting were observed, as exemplified by *R*^2^ values of 0.51, 0.86 and 0.63, respectively. This confirms that our biomarker product score remains valid in syngeneic and orthotopic tumours in immunocompetent mice.

**Fig. 6:**
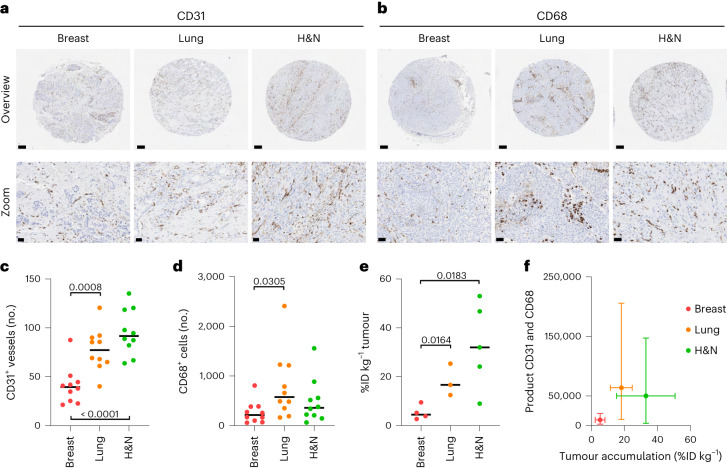
Validation of histopathological biomarker scoring in patient datasets. **a**,**b**, Representative DAB stainings of blood vessels (**a**) and TAM (**b**) in tumour tissues obtained from patients with breast, lung and head and neck (H&N) cancer (all data in this figurre are based on tumour resections, and the data based on biopsies are shown in Supplementary Fig. 7). **c**,**d**, Quantification of blood vessels (**c**) and TAM (**d**) in ten patient samples for each tumour type (no., number per field of view; significance is indicated in *P* values based on Student’s *t*-test). **e**, Tumour accumulation of ^111^In-labelled PEGylated liposomes in patients with breast, lung and head and neck (H&N) cancer (in percentage of the injected dose per kilogram tumour). The data are replotted based on the work in ref. ^27^ (significance is indicated in *P* values based on Student’s *t*-test). **f**, Means of blood vessel and TAM product scores plotted against means of liposome tumour targeting, showing that biomarker product scoring correctly identifies breast cancers as poorly nanomedicine accumulating lesions. The error bars indicate the distribution of %ID and product score values (standard deviations on the *x*-axis and minima and maxima on the *y*-axis; *n* = 3–10 as it is based on the means of **c**, **d** and **e**).

Next, we aligned our biomarker product score with the most comprehensive clinical dataset available on nanomedicine tumour targeting in patients^27^. In this study, the researchers used ^111^In-labelled PEGylated liposomes and quantitative SPECT imaging to assess nanomedicine tumour accumulation in 17 patients with different type of tumour^27^. For the most prevalent tumour types included, that is, ductal breast cancer, squamous cell carcinoma of the lung and squamous cell head and neck cancer, we collected matching tumour resection samples as well as primary tumour biopsies from the Biobank archive of the Institute of Pathology at RWTH Aachen University Hospital (Supplementary Table 5). Blood vessel (CD31^+^) and TAM (CD68^+^) density were analysed in ten different patient samples for each of the three cancer types, always in five different microarray sections for each individual tumour specimen. The expression levels and patterns of F4/80 and CD68 on TAM were demonstrated to be similar (Supplementary Fig. 6). Representative CD31 and CD68 stainings for breast, lung and head and neck cancer lesions are shown in Fig. 6a,b. Using QuPath software^28^, we quantified blood vessel and TAM density in these tumours and found that breast cancer typically presents with much lower levels of both tumour-tissue biomarkers as compared with lung and head and neck cancer (*P* < 0.001 and *P* < 0.0001 for blood vessels and *P* < 0.05 for TAM; Fig. 6c,d).

The liposome tumour targeting data from ref. ^27^ is replotted in Fig. 6e. In line with our rationale and reasoning, it can be seen that ductal breast cancer lesions in patients (5.3 ± 3.0 %ID kg^−1^) accumulate radiolabelled PEGylated liposomes significantly less well than lung (18.2 ± 6.6 %ID kg^−1^; *P* < 0.05) and head and neck (33.0 ± 17.6 %ID kg^−1^; *P* < 0.05) squamous cell carcinomas. When generating tumour-tissue biomarker product scores based on the number of blood vessels and TAM per tumour type and when plotting these product scores against the average level of liposome accumulation per tumour type, we found that breast cancers clustered in the lower left corner, thereby pinpointing them as true negatives (Fig. 6f). For the majority of lung and head and neck cancer lesions, the product scores were much higher than for breast cancer, thereby classifying them as true positives. In a final validation study, we also employed the original primary tumour biopsies for biomarker assessment. For the 30 patients samples initially included, 28 primary biopsies were available. As exemplified by Figure S7, the results obtained in biopsies are very similar to those obtained in resected tumour tissues, again clearly identifying ductal breast cancers as poorly accumulating lesions. Thereby, they not only confirm the robustness of our approach but also showcase its clinical translatability. Altogether, these findings provide compelling proof-of-concept for the use of tumour blood vessels and TAM as tissue biomarkers for predicting nanomedicine tumour targeting.

## Discussion

We present a histopathological biomarker score to help realize patient stratification in clinical trials of cancer nanomedicine. Being able to stratify patients into low versus high ‘nanomedicine accumulators’ via a robust and straightforward protocol would enable the exclusion of patients unlikely to respond to the therapy. This could mark a step change towards promoting the clinical translation of cancer nanomedicines, via an approach that considers disease heterogeneity.

Initially, in a training panel of three tumour models, we quantified PHPMA tumour accumulation over time and correlated it with 23 microenvironment features. We employed GTB-based machine learning to identify the features most prominently contributing to nanomedicine target-site accumulation. GTB is a hypothesis-free approach and, thus, highly suitable for this purpose^23^. Five of six features top-ranked for importance were related to the tumour vasculature, with the sixth feature being macrophage density. These findings are in line with previous studies connecting tumour blood vessels and TAM with nanomedicine tumour targeting^17,29–32^. They are also consistent with the notions that there generally is a good correlation between blood vessel density and TAM density in a broad range of tumour models, partly substantiating the intimate relationship between these two features^33^. It is not surprising that tumour blood vessels are identified as a key biomarker, given their presence throughout the tumour mass and their direct role in the circulation, distribution and tumour-directed delivery of drugs and drug delivery systems^17,29–31^. It is also reasonable that TAM show high feature importance, as they have on multiple previous occasions been linked to nanomedicine tumour accumulation, nanoparticle redistribution within tumours, formation of local drug delivery depots and therapy treatment outcomes^8,20,32,34,35^.

The biomarker performance of our tumour blood vessel and TAM product score was validated in three syngeneic tumour models in immunocompetent mice, as well as in ten CDX and PDX xenograft tumour models in immune-deficient mice. The latter were selected to represent a wide and clinically relevant range of tumour aetiologies, microenvironment architectures and liposome accumulation patterns. This extensive validation was deemed necessary to enable a robust assessment of tumour-tissue biomarker correlation with nanomedicine delivery and to avoid any tumour model-, host- or nanocarrier-based bias. In the ten xenograft models, we consistently observed—with one exception (Calu-3)—that PDX models always more efficiently accumulated liposomal DXR than CDX models (Fig. 4a–c). In agreement with this, we noticed—with two exceptions (Calu-3 for blood vessels and E35CR for macrophages)—that the staining density of CD31 and F4/80 was always higher in PDX models (Fig. 4d–g), corroborating the positive correlation between these two biomarker features and tumour-targeted drug delivery.

Via our biomarker product score, we were able to identify tumours with inefficient nanomedicine delivery, correctly assigning nine out of ten PDX and CDX tumour models to either low or high levels of accumulation (with an AUROC of 0.91; Fig. 5e). This notion supports our primary hypothesis that nanomedicine tumour targeting can be predicted on the basis of tumour-tissue biomarkers. E35CR was the only tumour model for which stratification did not work, turning out as a false negative, with high levels of liposome accumulation (Fig. 4a–c), in spite of a low tumour-tissue biomarker product score (Figs. 4d,g and 5c,d).

Our biomarker product score primarily considers bulk accumulation as a key driver of efficacy. Further granularity at the level of specific cell uptake in the tumour microenvironment was not required to differentiate between low and high nanomedicine accumulators. It is important to bear in mind in this regard that traditional chemotherapy-loaded nanodrugs, such as Doxil, are increasingly widely accepted to work indirectly, via initial uptake in and processing by TAM and via subsequent liberation of encapsulated small molecule drugs, which kill surrounding cancer cells^8,20,32,34,35^. Biomarker information on nanomedicine uptake by cancer cells consequently seems less important for predicting tumour targeting and therapy efficacy than biomarker information on TAM density. An important exception here are drugs that have to be delivered intracellularly into cancer cells to exert therapeutic effects, for example, siRNA directed against an oncogene^36^. For such agents, uptake by TAM is not helpful, because liberated siRNA will not be able to enter cancer cells on its own (unlike, for example, DXR), and also because oncogene knockdown in TAM will not have a therapeutic effect. Ligand-mediated active targeting may help in such situations, beneficially shifting the balance from predominant delivery to TAM towards more efficient delivery to and into cancer cells^37,38^. Our tumour microenvironment biomarkers determining tumour-directed drug delivery complement tissue biomarkers already available in the clinic for patient stratification in case of actively targeted therapeutics (for example, HER2 staining in case of intended treatment with the HER2-targeted antibody–drug conjugates adotrastuzumab emtansine (Kadcyla) or trastuzumab deruxtecan (Enhertu)). Since both passively and actively targeted (nano)therapeutics rely on pathophysiological and physical properties of the tumour microenvironment for transportation to, distribution in and efficacy against malignant lesions, the use tumour-tissue biomarker information specifically related to the drug delivery process seems to be valuable not only for guiding patient stratification but also for furthering our clinical understanding of tumour-targeted drug delivery and the opportunities and limitations thereof^39^.

The overall aim of this study is to establish a robust and straightforward protocol for patient stratification in clinical trials of cancer nanomedicines. For obvious reasons, the protocol should eventually predict treatment outcome rather than tumour accumulation of the nanomedicine (even though multiple previous preclinical and clinical papers have convincingly shown that the tumour accumulation of nanomedicines correlates well with treatment outcomes^8–14^). A key limitation of the study is that, owing to ethical and practical constraints, we were not able to correlate the tumour-tissue biomarker score with nanomedicine treatment efficacy, but could only robustly demonstrate a correlation between biomarker product score and nanomedicine tumour accumulation. Ethical issues include the facts that mouse tumours cannot be properly biopsied and that sampling of tumours in patients cannot easily be done solely for scientific purposes. Practical reasons include the notions that performing retrospective analyses is not helpful (even if both tumour tissue and response data are available, because cancer nanomedicines are hardly ever used as monotherapies) and that prospective clinical trials are only feasible when coupled to the development and translation of a novel nanomedicine formulation. We are confident that in future prospective trials, histopathological biomarkers in tumour tissue will be valuable and easily implementable to help identify the right patients for inclusion in study arms, thereby potentially marking an important step forward towards promoting the translational prospects of cancer nanomedicines.

## Methods

### Synthesis and characterization of polymeric and liposomal nanomedicines

Fluorophore-labelled PHPMA polymers were used to evaluate the biodistribution via in vivo and ex vivo optical imaging. Polymer synthesis was performed as in ref. ^40^. They were characterized via size-exclusion chromatography and presented with a hydrodynamic radius of 4.1 nm and a polydispersity index of 1.7. The dye content was measured via ultraviolet/visible light spectrophotometry and found to be 2.1% w/w for Atto488 and 1.6% w/w for Dye750.

Fluorophore-labelled liposomes were used to evaluate intratumoural distribution via fluorescence microscopy. For this purpose, DOPC/CHOL/mPEG2000-DSPE (50:45:5 mol/mol) liposomes labelled with the dye DiI (0.5 mM) were purchased (FormuMax). DXR-loaded liposomes were used to evaluate tumour accumulation via liquid chromatography–mass spectrometry. To prepare DXR-loaded liposomes, 100 nm (95–120 nm) HSPC/CHOL/mPEG2000-DSPE (50:45:5 mol%) liposomes containing a 250 mM ammonium sulfate gradient were purchased from FormuMax (product code F20204AB). DXR was loaded via the ammonium sulfate gradient method during a 60 min incubation, and the unloaded drug was separated using a Sephadex G-50 column to obtain a formulation similar to the commercially available liposomal formulation Doxil/Caelyx. The final DXR concentration was determined using DXR absorbance at 480 nm measured against a standard curve and was typically ~1.3 mg ml^−1^. The liposomes were diluted with saline to 0.3 mg DXR ml^−1^ or 0.5 mg DXR ml^−1^ immediately before dosing.

### In vivo experiments

All animal experiments were approved by the responsible governmental review committees on animal care. All work conducted in the UK adhered to the Animal Scientific Procedures Act 1986 and complied with the Global Bioethics Policy. All experiments were detailed in approved project licenses outlining the exact type of research performed and initially went through internal ethical review process, followed by assessment and approval by the LANUV (Germany) and Home Office (United Kingdom). All experiments followed the principles of good statistical practice, as well as the PREPARE and ARRIVE guidelines. AstraZeneca is a signatory to the Concordat on Openness on Animal Research in the United Kingdom.

### Tumour accumulation of PHPMA polymers

The biodistribution and tumour accumulation of polymeric nanocarriers was studied in 14 CD1 nude mice (6–8 weeks old) and in four BALB/c mice (6–8 weeks old). The animal housing was done with food and water ad libitum, controlled light cycles and in individually ventilated cages. The tumours were inoculated in the right flank (or orthotopically for 4T1) under continuous inhalation anaesthesia (using 2% v/v isoflurane; A431 4 × 10^6^ cells; MLS 5 × 10^6^ cells; CT26 1 × 10^6^ cells; 4T1 2.5 × 10^4^ cells each in 100 µl medium). Upon reaching a size of 7 mm, the tumour accumulation experiment was initiated, and the food was changed 3 days in advance to a fluorescent background minimizing, chlorophyll-free diet (ssniff Spezialdiäten GmbH). The biodistribution of the polymers was monitored at 0.25, 4, 24 and 72 h post i.v. injection via CT–FMT (PerkinElmer), using a custom-made mouse bed. CT–FMT imaging and image analysis were performed as described previously^41^. After the last CT–FMT scan, the animals received an i.v. injection of rhodamine lectin (Vector Laboratories) to stain functional blood vessels, upon which they were killed. The excised tumours were subsequently scanned using FRI (PerkinElmer) and embedded in TissueTek O.C.T. (O.C.T., optimal cutting temperature; Sakura Finetek Europe).

### Tumour accumulation of liposomes

The tumour accumulation and intratumoural distribution of liposomes was studied in 12 CDX and PDX models in C57BL/6J, nude and SCID mice. Mice were housed in controlled conditions in accordance with the local and national guidelines. Access to food and water was ad libitum. Studies with Hep55-1.C tumour models (subcutaneous and orthotopic) were completed at University Hospital RWTH Aachen (Germany).

Studies with PDX models OVFX899, LXFE2257, CFX1297 and RXF423 were completed at Oncotest (now Charles River, Germany). Studies with PDX models E77 and E35CR^42^ and CDX models Calu-3, Calu-6, A549 and SW620 were completed at AstraZeneca (United Kingdom). The Hep55-1.C cells were implanted either into the left flank (subcutaneously) or into the left liver lobe under sterile conditions (orthotopically) at 2 × 10^6^ cells in 50% Matrigel. The Calu-3 cells were implanted at 8.8 × 10^6^ cells per mouse in 30% Matrigel, the A549 cells were implanted at 5 × 10^6^ cells in 50% Matrigel and Calu-6 cells and SW620 cells were implanted at 1 × 10^6^ cells in 50% Matrigel. The PDX models were implanted subcutaneously as 3 × 3 mm fragments (Supplementary Table 3). For liposome experiments, dosing was initiated once tumours reached an average volume of 500 mm^3^, following randomization to achieve a similar mean initial volume distribution across treatment groups. In all studies, mice were administered a single i.v. slow bolus injection of liposomes via the lateral tail vein.

To compare liposome distribution across the models, DiI-labelled DOPC/CHOL/mPEG2000-DSPE liposomes were diluted in physiological saline and dosed i.v. at 15 mg kg^−1^ (total lipid concentration) to tumour-bearing mice (*n* = 3 per model). This concentration matches the lipid dose administered to mice in experiments using DXR-loaded liposomes. At 24 h post-dose, mice were humanely killed, and the tumours were excised and immediately snap-frozen in liquid nitrogen. The DiI signal was subsequently visualized in tumour sections that had been stained for CD31 via immunofluorescence (see ‘Tissue staining and microscopy analysis of murine tumours’ section).

To compare liposome accumulation and retention across the models, DXR-loaded liposomes (3 or 5 mg DXR kg) were diluted in physiological saline and administered as a single i.v. bolus of 3 or 5 mg kg^−1^ (DXR concentration) to tumour-bearing mice (*n* = 5 per model per timepoint). At various timepoints post-dose of vehicle or liposomes, mice were humanely killed via a schedule 1 method with secondary confirmation performed before whole tumours were excised and immediately snap-frozen. The DXR concentration in tumours was subsequently measured via liquid chromatography–mass spectrometry against a standard curve in tumour homogenate. The DXR concentrations in tumour were subsequently normalized for dose to allow comparison between all models, whether dosed with 3 mg kg^−1^ or 5 mg kg^−1^, as necessitated due to strain differences in DXR tolerability. Untreated control tumours were also resected and immediately formalin-fixed and paraffin-embedded following standard protocols. These samples were used for comparison of TME biomarkers at baseline across models via DAB staining.

### Tissue staining and microscopy analysis of murine tumours

Immunofluorescent stainings were performed on 8 µm tumour-tissue cryosections, upon fixation with 80% v/v methanol aqueous solution for 5 min and then 20 min of −20 °C acetone. Primary and secondary antibodies (and concentrations) are listed in Supplementary Table 4, and incubations were done either for 1 h at room temperature or overnight at 4 °C for primary antibodies and 1 h at room temperature for secondary antibodies. The image acquisition was done via the Axio Imager M2 microscope (Carl Zeiss AG), and four images of three sections per tumour were analysed using FiJi software^43^.

Immunofluorescent staining for CD31 was also performed on tumour sections from mice bearing CDX or PDX tumours and dosed with DiI-labelled liposomes. Frozen cryosections (15 µm thick) were air dried at room temperature for 20 min and blocked with 20% normal goat serum (Sigma) for 20 min at room temperature. The solution was blown off gently and then the sections were incubated with 1:50 CD31-488 (clone MEC13.3; Biolegend) for 60 min at room temperature. Sections were gently rinsed with water and cover-slipped using 4,6-diamidino-2-phenylindole-containing mountant (Thermofisher). Image acquisition for DiI and Alexa488 signal was performed using a Mirax Scan (Zeiss).

To characterize the baseline expression levels of CD31 and F4/80 across the 10 CDX and PDX models, FFPE sections (4 µm thick) of untreated tumours (*n* = 5 per model) were stained individually for CD31 and F4/80 via DAB staining (see Supplementary Table 4 for specifications).

### Tissue staining and microscopy analysis of human tumour samples

Ethical approval for analysis of human tumour samples was obtained by the ethics committee of the University Hospital RWTH Aachen (CTC-A no. 21-359 and EK no. 22-294). The human cancer tissue samples were dehydrated and paraffin-embedded according to a routine diagnostic protocol. For the analysis of the tumour macrophages and blood vessels, samples from breast (topography code C50 and International Classification of Diseases for Oncology (ICD-O) code 8500/3 infiltrating duct carcinoma, NOS), lung (topography code C34 and ICD-O code 8070/3 squamous cell carcinoma, not otherwise specified) and head and neck (topography codes C02-C13, C32 and C44 and ICD-O code 8070/3 squamous cell carcinoma, not otherwise specified) were identified (see Supplementary Table 5 for sample details) and retrieved from the routine diagnostic archive from the Institute of Pathology at RWTH Aachen University Hospital. In accordance with patients’ characteristics provided in ref. ^27^, tumours with a locally advanced T3–T4 tumour stage according to the TNM classification were selected (Supplementary Table 5). The patients had not undergone neoadjuvant chemotherapy before surgery.

From formalin-fixed paraffin-embedded donor tumour-tissue specimens, five to six punches with a diameter of 2 mm were obtained with a tissue microarray device (TMArrayer, Pathology Devices, product number 02-11-0016) for each individual lesion, and they were transferred to an acceptor paraffin block. Ten breast, lung and head and neck cancer samples from ten different patients were arrayed on one acceptor block. After incubation of the acceptor blocks in an oven at 37 °C overnight, the blocks were cut into 4 µm cuts and mounted on coated microscope slides (Dako, K8020). Next, the sections were deparaffinized and rehydrated, and target retrieval was performed (Dako, K8005) in a pre-treatment module (PT-Link, Dako, at pH 9 for CD31 staining and at pH 6.1 for CD68 staining). Using an autostainer (Thermo Fisher Scientific, A80500003), the slides were incubated with endogenous peroxidase blocking solution for 5 min, followed by anti-human CD31 antibody or anti-human CD68 antibody (see Supplementary Table 4 for specifications) for 30 min. Next, slides were incubated with goat secondary antibody molecules against mouse immunoglobulins conjugated to a peroxidase-labelled polymer chain (Dako EnVision FLEX, K8000) for 30 min. The antigen–antibody–polymer complex was visualized with DAB + chromogen (Dako, K8002) for 10 min. The counterstaining was performed with haematoxylin (Dako, K8008) for 5 min. Finally, the slides were covered with cover-slipping film (Sakura, 6132 Prisma). The immunohistochemical DAB stainings were analysed via inForm (for tissue segmentation, Akoya BioSciences) or QuPath (for positive cell detection^28^).

### Gradient tree boosting

The importance of all 23 histopathological features was subsequently studied using GTB-based machine learning. GTB is a supervised machine learning technique building predictive regression models based on a set of decision trees^21–23^. Other machine learning methods, such as support vector machine modelling [28], would also be suitable for performing the tasks investigated here. We chose to use GTB because it enables the ranking of feature importance, thereby promoting the identification of individual features’ relative contribution to improved nanomedicine tumour accumulation. In GTB, every decision tree is established as a chain of simple comparisons with a binary outcome. The ensemble is trained in an additive manner, that is, every newly added decision tree corrects the results of the previous present decision trees. GTB accepts arbitrary input features and intrinsically handles partially missing data during training and prediction^44^. Important hyperparameters of GTB models, that is, parameters set before the model training, are the maximum depth, the number of decision trees and the learning rate^45^. The maximum depth denotes the maximum number of comparisons within a single decision tree. The learning rate is only essential during model training and weights the influence of the previous ensemble when adding the following decision tree. Within the model used here, the number of decision trees was set to ten and the maximum depth to eight. The python environment and GTB training and analysis are described in Supplementary Information. Trained GTB models allow insights into their prediction process as the individual decision trees can be easily followed and the used features are recognizable. This allows calculating the feature importance by calculating the distribution and occurrence of the features in the comparisons, measuring the relevance of every individual input feature for the whole GTB ensemble.

### Statistical analysis

The statistical analyses were performed using GraphPad Prism 9. The results are either plotted as values of individual images or as averages per animal and/or tumour type, extended by means and standard deviations, respectively. When two groups were compared, Student’s *t*-tests were used. When multiple groups were compared, one-way ANOVA was used. Details on the tests employed are provided in figure legends. The GTB results were evaluated using the Python environment^45^.

### Reporting summary

Further information on research design is available in the Nature Portfolio Reporting Summary linked to this article.

## Supplementary information

Supplementary Information Supplementary Figures, Tables and Methods.

Reporting Summary

## Data Availability

The main data supporting the results of this study are available within the paper and its Supplementary Information. Whole-slide images of human tumour sections cannot be made publicly available owing to regulatory constraints. Models and data will be made available to interested research partners on reasonable request to the corresponding author; the prerequisite for this is a data-transfer agreement, approved by the legal departments of the requesting researcher and by all legal departments of the institutions that provided data for the study, as well as an ethics clearance.

## Code availability

The code of the GTB model is available from the corresponding author on reasonable request. A description of the workflow is provided in Supplementary Information.
