# Supplementary material for: Histopathological biomarkers for predicting the tumour accumulation of nanomedicines
Source: Nat Biomed Eng. 2024 Apr 8;8(11):1366–78. doi: 10.1038/s41551-024-01197-4 (PMC7616664; doi:10.1038/s41551-024-01197-4)
Supplement: Supplementary file 1 — Supplementary Figures, Tables and Methods. [file 41551_2024_1197_MOESM1_ESM.pdf]

nature biomedical engineering

Article

https://doi.org/10.1038/s41551-024-01197-4

Histopathological biomarkers for predicting the tumour accumulation of nanomedicines

In the format provided by the authors and unedited

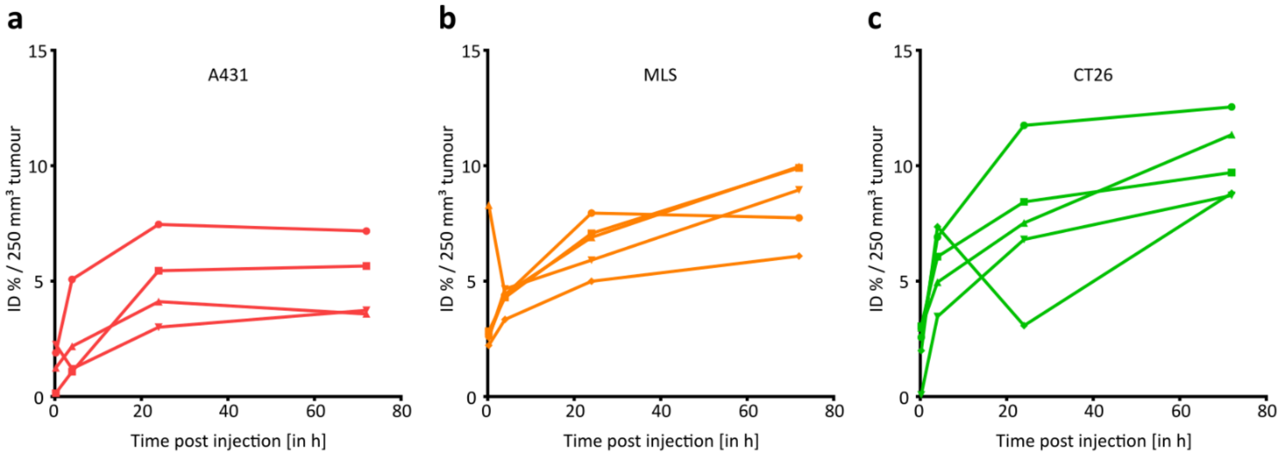

Supplementary Fig. 1 l Tumour accumulation of PHPMA nanocarriers over time. CT-FLT imaging was employed to kinetically monitor the tumour accumulation of 67 kDa-sized DY750-labeled PHPMA in individual animals in three different tumour models. The results show that polymer accumulation in A431 tumours plateaued from 24 h onwards, while in MLS and CT26 tumours, the concentrations of the nanocarrier still slightly increased from 24 to 72 h post i.v. injection.

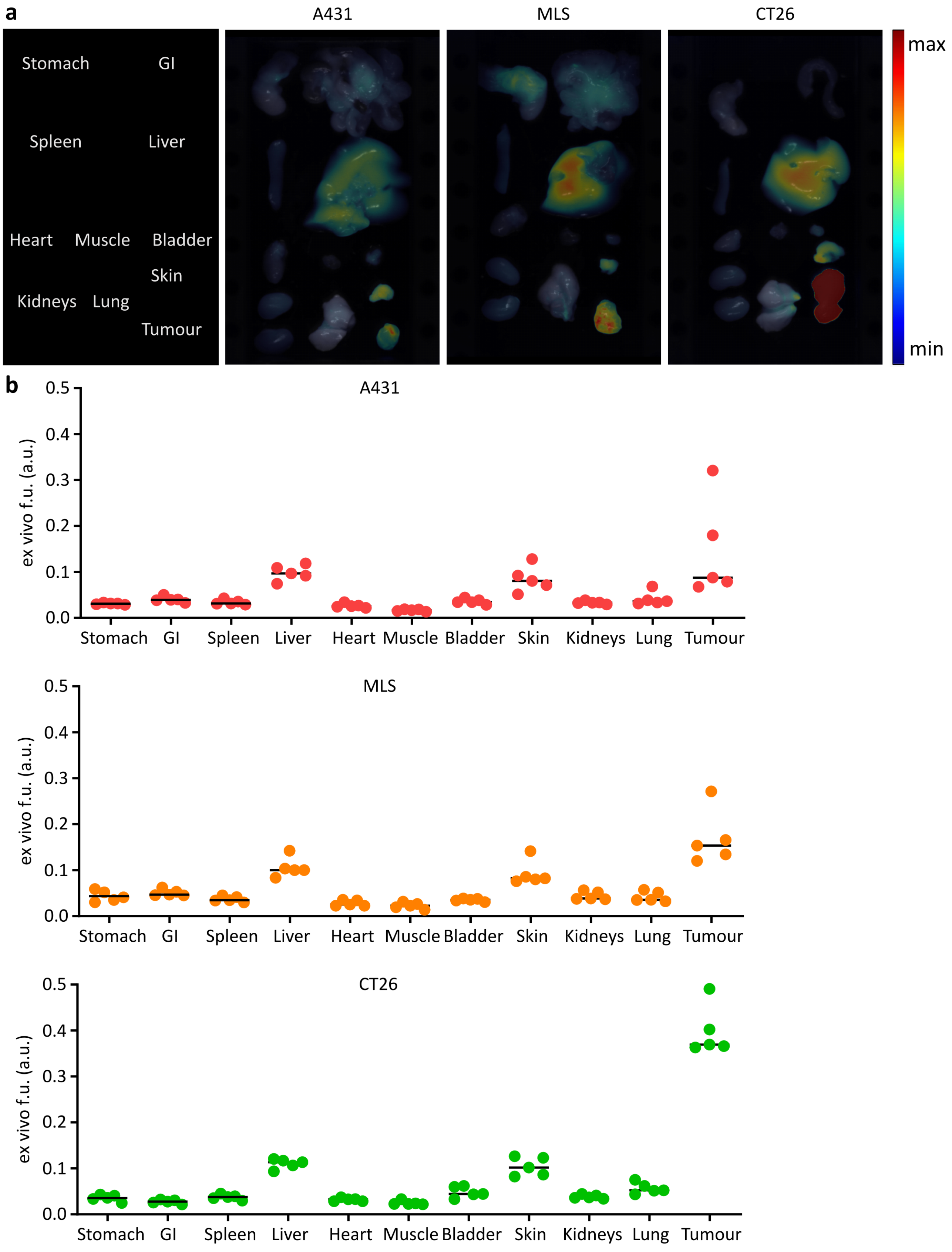

Supplementary Fig. 2 | Ex vivo analysis of PHPMA biodistribution. a, Fluorescence reflectance imaging (FRI) analysis displaying the biodistribution and tumour accumulation of 67 kDa DY750-labeled PHPMA in 3 different tumour models at 72 h post i.v. injection. **b**, Fluorescence signal analysis for tumours and organs, demonstrating efficient tumour vs. organ targeting as well as differential tumour accumulation in A431, MLS and CT26 tumours.

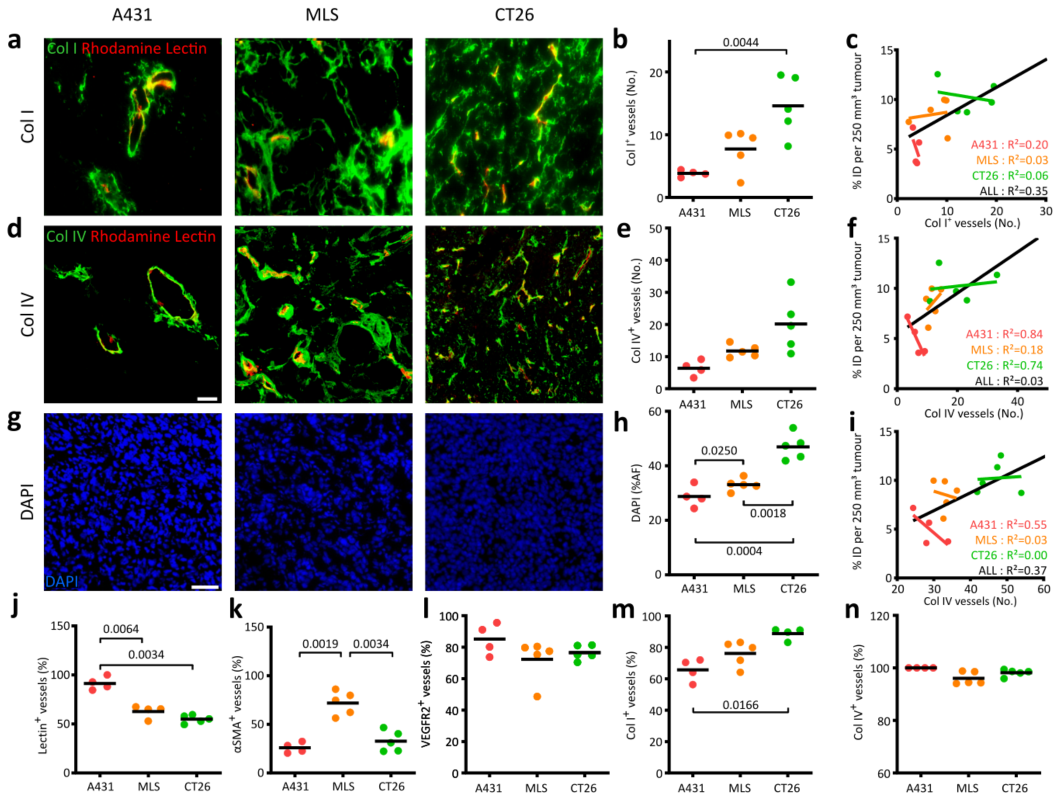

Supplementary Fig. 3 I Tumour microenvironment characterization and correlation with nanocarrier accumulation. a, d, g, Immunofluorescence stainings for collagen I (Col I), collagen IV (Col IV) and nuclei (DAPI) in A431, MLS and CT26 tumours. **b, e, h**, Quantification of the immunofluorescence images. Black bars indicate means. **c, f, i**, Correlation of polymeric nanocarrier tumour accumulation at 72 h post i.v. injection with tumour tissue biomarker features. Trendlines are shown per tumour model (colour-coded) and for all tumours together (black). R^2 values indicate the coefficient of determination and reflect the goodness of fit. **j-n**, Evaluation of additional biomarker features relying on double-staining with tumour blood vessels (CD31). Plotted are the percentages of lectin+, αSMA+, VEGFR2+, Col I+ and Col IV+ vessels in the three tumour models employed. All indicated p values are based on Student's t-test.

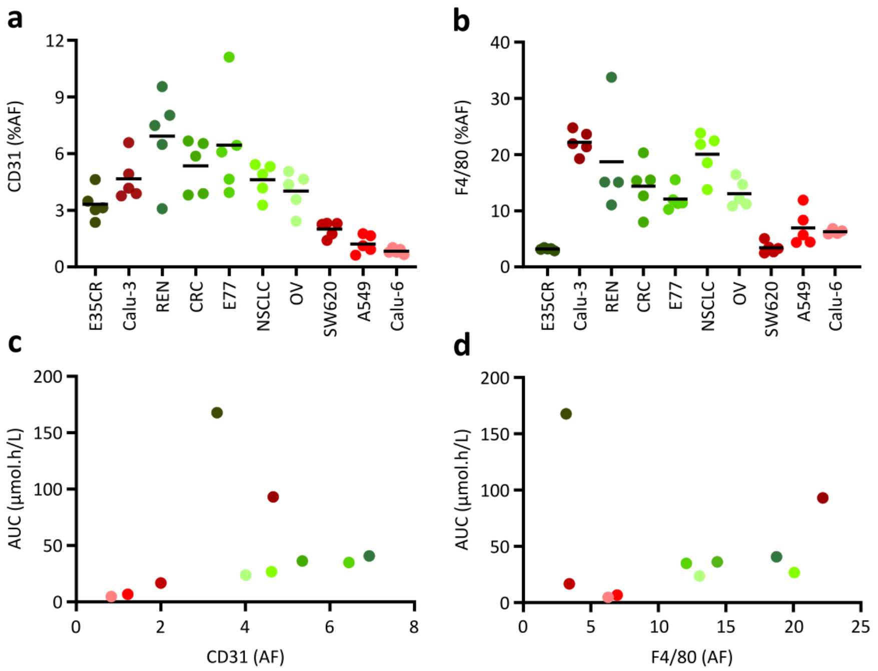

Supplementary Fig. 4 I Correlation of CD31 and F4/80 area fraction with liposome tumour accumulation. a, b, In addition to vessel and macrophage number, also the area fraction of CD31- and F4/80-positive structures were quantified in the DAB stainings of the 10 tumour models. Black bars indicate means. **c, d**, Correlation of CD31 and F4/80 area fraction with the tumour accumulation of liposomal doxorubicin over time ($AUC_{0\text{-}120}$). Note that E35CR is an outlier, and that overall, there is a decent correlation between blood vessel and macrophage density and liposomal doxorubicin accumulation.

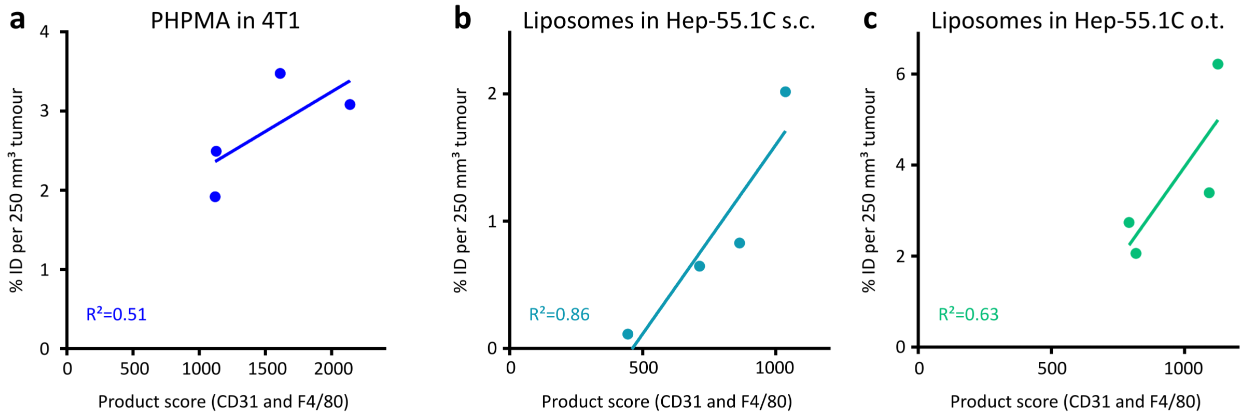

Supplementary Fig. 5 l Nanocarrier tumour accumulation in immune-competent mouse models. a, 4T1 tumour-bearing BALC/c mice were injected with PHPMA polymers and tumour accumulation was plotted against the product score of tumour blood vessels and TAM. **b-c**, Hep-55.1C tumour cells were inoculated subcutaneously (**b**) or orthotopically (**c**) in immune-competent C57BL/6J mice, and the tumour accumulation of PEGylated liposomes was plotted against the product score of tumour blood vessels and TAM. In all three syngeneic tumour models in immune-competent mice, a good correlation was observed between our biomarker product score and nanomedicine tumour accumulation.

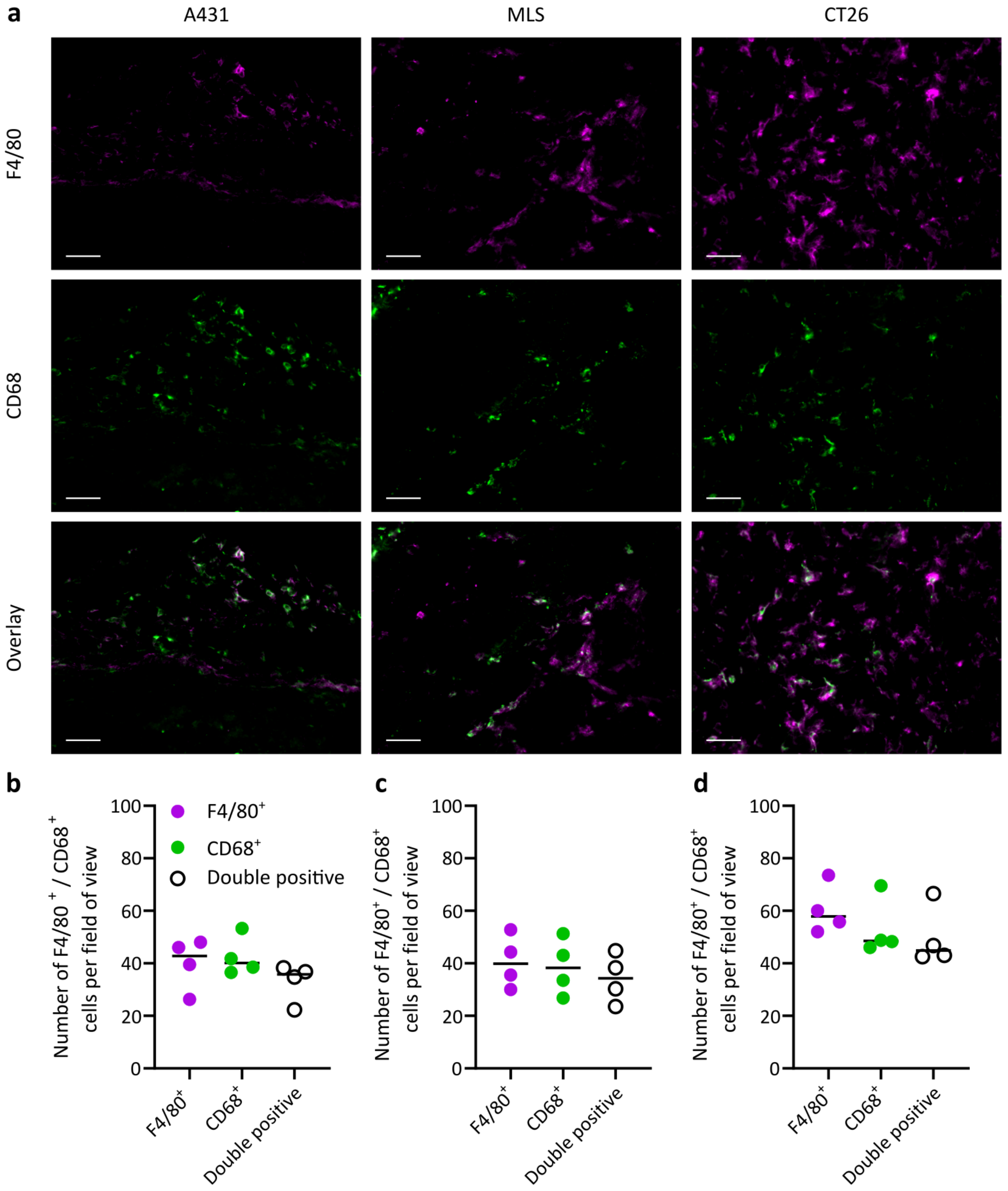

Supplementary Fig. 6 | Co-staining of tumour-associated macrophages with F4/80 and CD68 antibodies. a, Fluorescence microscopy analysis of A431, MLS, and CT26 tumours in which TAM were co-stained for F4/80 and CD68. Scale bars indicate 50 μm. **b-d,** The number of cells positive for F4/80, for CD68 and double-positive for both F4/80 and CD68 were counted per field of view. Values represent the average of n=5 different fields of view for each individual tumour. The images and quantification show that there is high congruence between F4/80 and CD68 staining.

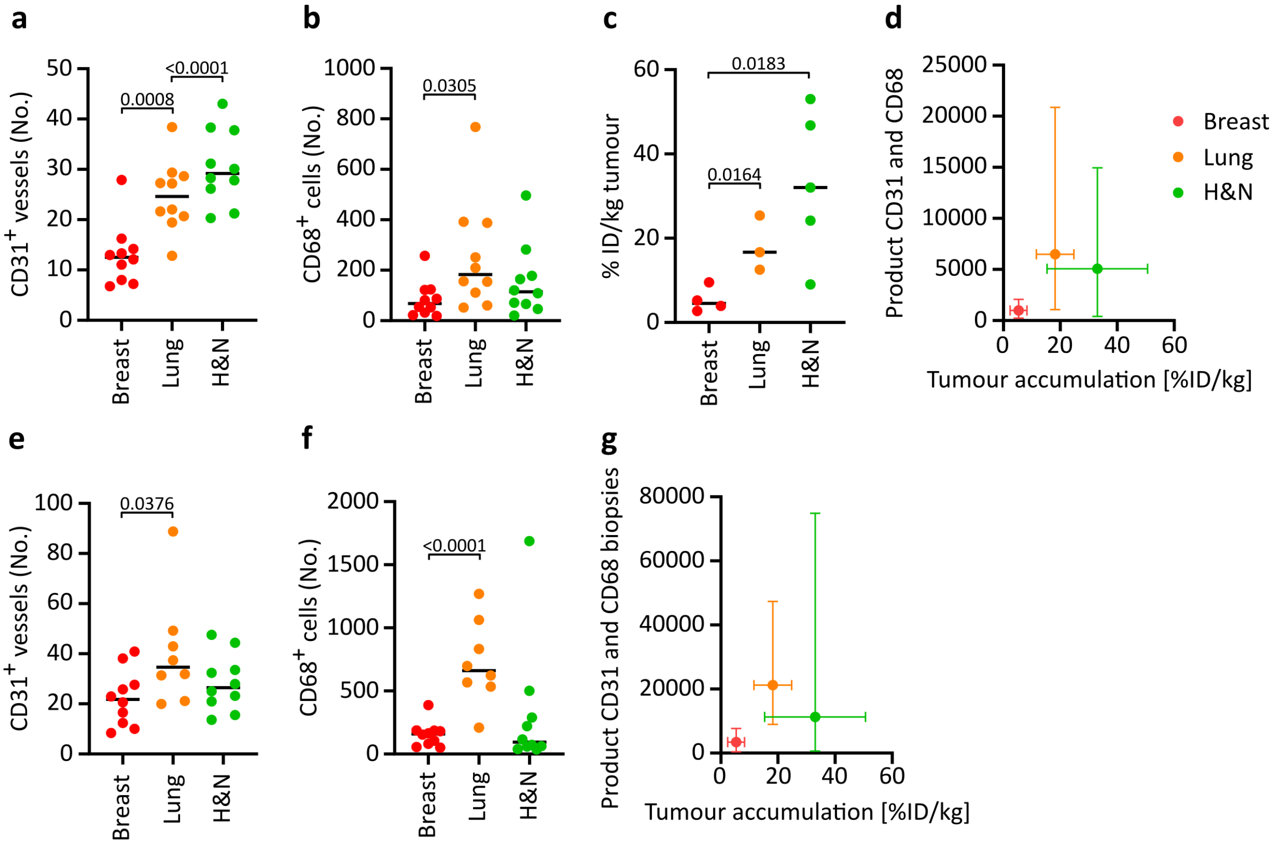

Supplementary Fig. 7 l Comparison of biomarker assessment and product score performance in resected tumour tissue specimens vs. primary tumour biopsies. a-d, Product score performance in tumour resections. Data is replotted from main manuscript Fig. 7c-f, but now normalized to an area of 1 cm². Blood vessel counts are shown in panel (**a**) TAM counts in (**b**) liposome tumour accumulation in (**c**) (from Harrington et al. [24]), and CD31 and CD68 product scores versus liposome accumulation in (**d**). **e-f**, Quantification of blood vessel (**e**) and TAM (**f**) counts in corresponding tumour biopsies from the same patients. **g**, Means of CD31 and CD68 product scores in biopsies plotted against the means of liposome tumour targeting, exemplifying that also in biopsies, poorly accumulating tumours can be identified using biomarker assessment. Error bars indicate the distribution of product scores and tumour accumulation values (minima and maxima on the y-axis, standard deviations on the x-axis; indicated p values are based on Student's t-test).

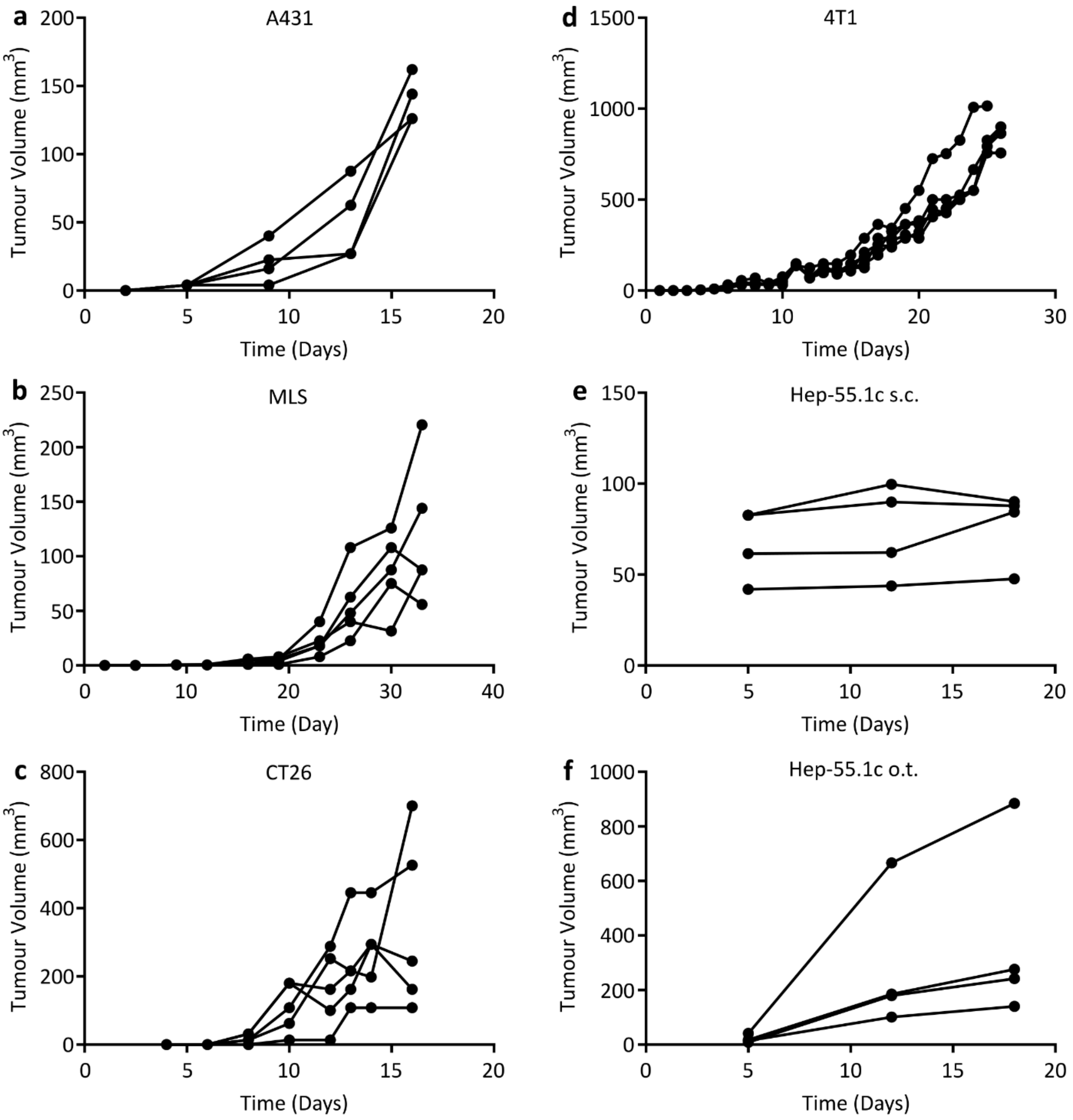

Supplementary Fig. 8 | Tumour growth of preclinical tumour models. a-f, Tumour volume calculations are based on caliper measurements or on CT imaging (**e,f**).

Supplementary Table 1 I Overview of tumour tissue biomarkers.

Category	Antigen	Indicative for	Analysed Biomarker
Vasculature	CD31	Blood vessels	Number (No.)
			Area Fraction (AF)
	Lectin	Perfused Vessels	Number (No.)
			Area Fraction (AF)
			Ratio of pos. vessels
	VEGFR2	Angiogenesis	Number (No.)
			Area Fraction (AF)
			Ratio of pos. vessels
	LYVE-1	Lymphatic vessels	Number (No.)
			Area Fraction (AF)
Stroma	□SMA	Vessel maturity	Number (No.)
			Area Fraction (AF)
			Ratio of pos. vessels
	Col I	Extracellular Matrix	Area Fraction (AF)
			Number (No.) of pos. Vessels
			Ratio of pos. Vessels
	Col IV	Vessel support/basal lamina	Area Fraction (AF)
			Number (No.) of pos. Vessels
			Ratio of pos. vessels
Cells	F4/80	Macrophages	Area Fraction (AF)
			Number (No.)
	DAPI	Nuclei	Area Fraction (AF)
			Number (No.)

Supplementary Table 2 I Time post-implantation required for tumours to reach experimental size.

Tumour Model	Days to reach 0.5 cm^3
E35CR	43
Calu-3	45
REN (RXF423)	48
CRC (CXF1297)	21
E77	47
NSCLC (LXFE2257)	32
OV (OVFX899)	28
SW620	15
A549	21
Calu-6	29

Supplementary Table 3 I Details of preclinical tumour models.

Model	Mouse Sex and Strain	Mouse Supplier	Age / Weight at Implantation	Implant Conditions
A431<br>MLS<br>CT26	Female CD1-nude	Charles River	6-8 weeks	Cell suspension in medium. Right flank s.c. under anesthesia.
4T1	Female BALB/c	Charles River	6-8 weeks	Cell suspension in medium.<br>Mammary fat pad o.t. under anesthesia.
Hep55-1.C	Male C57BL/6J	Charles River	8-10 weeks	Cell suspension + Matrigel. Left flank s.c. or left liver lobe o.t. under anesthesia.
CRC (CXF1297)<br>NSCLC (LXFE2257) OV (OVFX899)<br>REN (RXF423)	Female NMRI nude	Harlan	4-6 weeks	3x3 mm fragment under anaesthesia.<br>Left flank s.c.
E35CR<br>E77	Male CB.17 SCID	Envigo UK	> 18 g	3x3 mm fragment under anaesthesia.<br>Left flank s.c.
Calu-3	Female CB.17 SCID	Envigo UK	> 18 g	Cell suspension + Matrigel. Left flank s.c.
A549<br>Calu-6<br>SW620	Female Hsd:Athymic Nude-Foxn1nu	Envigo UK	> 18 g	Cell suspension + Matrigel. Left flank s.c.

Supplementary Table 4 I List of antibodies.

**Primary antibodies**			
Antigen	Host	Dilution	Company & Catalogue number
Mouse CD31 (PECAM-1)	Rat	1:100	BD Biosciences # 553370
Mouse VEGFR2 extracellular domain	Goat	1:20	R&D Systems # AF644
Mouse F4/80 (wide range of Macrophages)	Rat	1:50	Bio-Rad # MCA497GA
Murine and human Collagen Type I	Rabbit	1:100	Novus Biologicals (NB600-408)
Mouse Collagen IV	Rabbit	1:100	Novotec # 20451 0.5ml
Mouse Smooth Muscle Actin (ASM-1)	Biotin	1:100	Progen # BK61501-1mg
Mouse LYVE-1	Rabbit	1:50	abcam # ab14917
Mouse CD68	Rabbit	1:100	abcam # ab125212
Human CD31 Clone JC70A	Mouse	ready to use	DAKO Code IR610
Human CD68 Clone PG-M1	Mouse	ready to use	DAKO Code GA613

**Secondary antibodies**			
Antigen	Conjugate	Dilution	Company & Catalogue number
Rat IgG (H+L)	Alexa Fluor 488	1:350	Dianova # 712-546-153
Rat IgG (H+L)	AMCA	1:50	Dianova # 712-155-153
Rat IgG (H+L)	Cy5	1:100	Dianova # 712-175-153
Rabbit IgG (H+L)	Alexa Fluor 488	1:500	Dianova # 711-546-152
Rabbit IgG (H+L)	AMCA	1:50	Dianova # 111-155-003
Goat IgG (H+L)	AMCA	1:50	Dianova # 705-155-147
Biotin	Cy2	1:200	Dianova # 016-220-084

Supplementary Table 5 | Details of patient tumour samples.

Sample ID	Sex	Cohort	ICD-O code	Topography code	TNM stage
BC1	Female	Breast	8500/3	C50	pT3pN1
BC2	Female	Breast	8500/3	C50	pT4pN3
BC3	Female	Breast	8500/3	C50	pT4pN2
BC4	Female	Breast	8500/3	C50	pT3pN2
BC5	Female	Breast	8500/3	C50	pT3pN0
BC6	Female	Breast	8500/3	C50	pT4pN3
BC7	Female	Breast	8500/3	C50	pT4pN2
BC8	Female	Breast	8500/3	C50	pT3pN3
BC9	Female	Breast	8500/3	C50	pT4pNX
BC10	Female	Breast	8500/3	C50	pT3pN1
HN1	Male	Head & neck	8070/3	C30	pT3pN0
HN2	Female	Head & neck	8070/3	C02	pT3pN1
HN3	Male	Head & neck	8070/3	C02	pT3pN2
HN4	Female	Head & neck	8070/3	C07	pT3pN1
HN5	Male	Head & neck	8070/3	C09	pT3pN1
HN6	Male	Head & neck	8070/3	C13	pT4pN0
HN7	Male	Head & neck	8070/3	C32	pT3pN0
HN8	Male	Head & neck	8070/3	C09	pT3pN1
HN9	Male	Head & neck	8070/3	C44	pT3pN1
HN10	Male	Head & neck	8070/3	C32	pT3pN0
LC1	Male	Lung	8070/3	C34	pT4pN1cM1
LC2	Male	Lung	8070/3	C34	pT3pN0
LC3	Male	Lung	8070/3	C34	pT3pN0
LC4	Male	Lung	8070/3	C34	pT3pN1
LC5	Female	Lung	8070/3	C34	pT3pN0
LC6	Male	Lung	8070/3	C34	pT3pN0
LC7	Female	Lung	8070/3	C34	pT3pN0
LC8	Male	Lung	8070/3	C34	pT3pN0
LC9	Male	Lung	8070/3	C34	pT3pN1
LC10	Male	Lung	8070/3	C34	pT3pN1

Supplementary methods

Gradient tree boosting
GTB is a supervised machine learning technique building predictive regression models based on a set of decision trees (1-3). Every decision tree is established as a chain of simple comparisons with a binary outcome. The ensemble is trained in an additive manner, i.e., every newly added decision tree corrects the results of the previous present decision trees. GTB accepts arbitrary input features and intrinsically handles partially missing data during training and prediction (4). Important hyperparameters of GTB models, i.e., parameters set before the model training, are the maximum depth, the number of decision trees, and the learning rate (5). The maximum depth denotes the maximum number of comparisons within a single decision tree. The learning rate is only essential during model training and weights the influence of the previous ensemble when adding the following decision tree. Trained GTB models allow insights into their prediction process as the individual decision trees can be easily followed and the used features are recognizable. This allows extracting the feature importance by calculating the distribution and occurrence of the features in the comparisons, measuring the relevance of every individual input feature for the whole GTB ensemble.

Python environment
The code is available on request and should run on all standard operating systems but was developed on a computer with an Intel processor and Windows 10. The usual runtime is in the range of 2-10 min.
For gradient tree boosting, a typical Python environment can be used (e.g., pip or Conda; here, Conda was used). The Conda-env is given in *env.txt* and should be installed in 10-15 minutes. Especially the XGBoost library version 1.4.2 was used.

Training and analysis procedure
The training and analysis consisted of two parts: First, a suitable set of hyperparameters was selected based on a grid search. Secondly, we identified the most relevant input features based on the feature importance (method "gain") and conducted an iterative feature reduction scheme.
In the first step, all available data was separated into training, validation, and test data sets (ratio 70:15:15). Then, a hyperparameter search for the maximum depth, number of decision trees as well as the learning rate was performed in the following ranges:

- Maximum depths: {3, 5, 7, 8, 9, 10}
- Number of decision trees: {10, 30, 50, 100}
- Learning rates: {0.1, 0.3}

The trained GTB models were tested for the R^2 and the mean absolute error, while the best suitable hyperparameters were selected based on the R^2. We chose the following hyperparameters set for the later model training, providing a well-predictive performance while keeping the models comparable small:

- Maximum depth: 8
- Number of decision tree: 10
- Learning rate: 0.1

All other hyperparameters were set to their default values. An overview as well as an explanation can be found in the official XGBoost documentation for the used library version 1.4.2. (6). These settings fully define the hyperparameter set of the employed GTB implementation.
In the second step, we analyzed the importance of the individual input features. The found hyperparameter set was fixed for further analysis. We chose a leave-one-out approach during model training and testing to ensure the best available database while separating training and test data. Thus, model training and evaluation were repeated for all available samples in the data set. After all GTB models were established required to ensure predicting the accumulation of all available samples, the feature importance of this model set was averaged and sorted. These results are shown and discussed in the main paper. In addition, we identified the least important feature, discarded it from the data set, and iteratively repeated this analysis. The listing of this "feature elimination" was consistent with the classical feature importance and thus not further discussed in the main work. As an advantage, feature elimination reduces the dimensionality of the feature space and allows testing for the models' stability for these reduced data sets. Thus, the analysis can provide hints for simplified application schemes acquiring only a subset of the discussed features.

References:

1. Kotsiantis SB. Decision trees: a recent overview. Artificial Intelligence Review. 2013;39:261-83.
2. Natekin A, Knoll A. Gradient boosting machines, a tutorial. Frontiers in neurorobotics. 2013;7:21.
3. Friedman JH. Greedy function approximation: a gradient boosting machine. Annals of statistics. 2001:1189-232.

4. Chen T, Guestrin C, editors. Xgboost: A scalable tree boosting system. Proceedings of the 22nd acm sigkdd international conference on knowledge discovery and data mining; 2016.
5. Müller F, Schug D, Hallen P, Grahe J, Schulz V. Gradient tree boosting-based positioning method for monolithic scintillator crystals in positron emission tomography. IEEE Transactions on Radiation and Plasma Medical Sciences. 2018;2(5):411-21.
6. https://xgboost.readthedocs.io/en/release_1.4.0/python/python_api.html
