# Supplementary material for: Histopathological biomarkers for predicting the tumour accumulation of nanomedicines
Source: Nat Biomed Eng. 2024 Apr 8;8(11):1366–78. doi: 10.1038/s41551-024-01197-4 (PMC7616664; doi:10.1038/s41551-024-01197-4)
Supplement: Supplementary file 2 — Reporting Summary [file 41551_2024_1197_MOESM2_ESM.pdf]

nature portfolio

Corresponding author(s): Twan Lammers

Last updated by author(s): Dec 15, 2023

Reporting Summary

Nature Portfolio wishes to improve the reproducibility of the work that we publish. This form provides structure for consistency and transparency in reporting. For further information on Nature Portfolio policies, see our Editorial Policies and the Editorial Policy Checklist.

Statistics

For all statistical analyses, confirm that the following items are present in the figure legend, table legend, main text, or Methods section.

n/a	Confirmed	
☐	☒	The exact sample size (*n*) for each experimental group/condition, given as a discrete number and unit of measurement
☐	☒	A statement on whether measurements were taken from distinct samples or whether the same sample was measured repeatedly
☐	☒	The statistical test(s) used AND whether they are one- or two-sided<br>*Only common tests should be described solely by name; describe more complex techniques in the Methods section.*
☒	☐	A description of all covariates tested
☐	☒	A description of any assumptions or corrections, such as tests of normality and adjustment for multiple comparisons
☐	☒	A full description of the statistical parameters including central tendency (e.g. means) or other basic estimates (e.g. regression coefficient) AND variation (e.g. standard deviation) or associated estimates of uncertainty (e.g. confidence intervals)
☐	☒	For null hypothesis testing, the test statistic (e.g. *F*, *t*, *r*) with confidence intervals, effect sizes, degrees of freedom and *P* value noted<br>*Give P values as exact values whenever suitable.*
☒	☐	For Bayesian analysis, information on the choice of priors and Markov chain Monte Carlo settings
☒	☐	For hierarchical and complex designs, identification of the appropriate level for tests and full reporting of outcomes
☐	☒	Estimates of effect sizes (e.g. Cohen's *d*, Pearson's *r*), indicating how they were calculated

Our web collection on statistics for biologists contains articles on many of the points above.

Software and code

Policy information about availability of computer code

Data collection	IMALYTICS Preclinical 2.1; FiJi ImageJ 2; inForm 2.1; QuPath 0.2.3.
Data analysis	XGBoost and Python; Graphpad Prism 5 and 9; Microsoft Excel.

For manuscripts utilizing custom algorithms or software that are central to the research but not yet described in published literature, software must be made available to editors and reviewers. We strongly encourage code deposition in a community repository (e.g. GitHub). See the Nature Portfolio guidelines for submitting code & software for further information.

Data

Policy information about availability of data

All manuscripts must include a data availability statement. This statement should provide the following information, where applicable:

- Accession codes, unique identifiers, or web links for publicly available datasets
- A description of any restrictions on data availability
- For clinical datasets or third party data, please ensure that the statement adheres to our policy

The main data supporting the results of this study are available within the paper and its supplementary information. Whole-slide images of human tumour sections cannot be made publicly available owing to regulatory constraints. Models and data will be made available to interested research partners on reasonable request to the corresponding author; the prerequisite for this is a data-transfer agreement, approved by the legal departments of the requesting researcher and by all legal departments of the institutions that provided data for the study, as well as an ethics clearance.

Research involving human participants, their data, or biological material

Policy information about studies with human participants or human data. See also policy information about sex, gender (identity/presentation), and sexual orientation and race, ethnicity and racism.

Reporting on sex and gender	The sex of patients was assessed during anamnesis (self-reported) with the tumour entity and stage as the most important inclusion criteria. We correlated our findings with data from Harrington et al. (Clin Cancer Res. 7, 243; 2001), who only unspecifically reported the sex of patients ("Seventeen patients (nine males and eight females"). As they included five patients with breast cancer, it is highly likely that five of eight female patients were allocated in the breast-cancer group, resulting in mainly male patients in the remaining cancer entities (lung, head and neck and glioma). Among our included patients, there was a comparable sex distribution with female patients with breast cancer and mainly male patients with lung and head and neck cancers. Taken together, sex was considered in the study design, yet gender was not.
Reporting on race, ethnicity, or other socially relevant groupings	We did not assess race, ethnicity or other socially relevant characteristics of the included patients as they were only included on the basis of their tumor type and stage.
Population characteristics	Participants of the retrospective study were included on the basis of their tumor type and stage, to allow a comparison with published data.
Recruitment	Samples were collected from the archive of the Institute of Pathology of RWTH Aachen University Hospital.
Ethics oversight	The study protocol was approved by the ethics commission of the medical faculty of RWTH Aachen University Hospital.

Note that full information on the approval of the study protocol must also be provided in the manuscript.

Field-specific reporting

Please select the one below that is the best fit for your research. If you are not sure, read the appropriate sections before making your selection.

☒ Life sciences ☐ Behavioural & social sciences ☐ Ecological, evolutionary & environmental sciences

For a reference copy of the document with all sections, see nature.com/documents/nr-reporting-summary-flat.pdf

Life sciences study design

All studies must disclose on these points even when the disclosure is negative.

Sample size	Sample sizes were determined from existing data on end-point assay variability.
Data exclusions	No data were excluded.
Replication	Biological replicates were used to ensure data reproducibility. Animal studies were completed only once.
Randomization	Animals were randomly assigned to the respective group. Randomization was completed via tumour volume for measurements of doxorubicin concentrations. Patient samples were included on the basis of tumor type and grade.
Blinding	The investigators were blinded to treatment during the analyses.

Reporting for specific materials, systems and methods

We require information from authors about some types of materials, experimental systems and methods used in many studies. Here, indicate whether each material, system or method listed is relevant to your study. If you are not sure if a list item applies to your research, read the appropriate section before selecting a response.

Materials & experimental systems

n/a	Involved in the study	
☐	☒	Antibodies
☐	☒	Eukaryotic cell lines
☒	☐	Palaeontology and archaeology
☐	☒	Animals and other organisms
☐	☒	Clinical data
☒	☐	Dual use research of concern
☒	☐	Plants

Methods

n/a	Involved in the study	
☒	☐	ChIP-seq
☒	☐	Flow cytometry
☒	☐	MRI-based neuroimaging

Antibodies

Antibodies used

Primary antibodies
Antigen Host Dilution Company & Catalogue number
Mouse CD31 (PECAM-1) Rat 1:100 BD Biosciences # 553370
Mouse VEGFR2 extracellular domain Goat 1:20 R&D Systems # AF644
Mouse F4/80 (wide range of Macrophages) Rat 1:50 Bio-Rad # MCA497GA
Murine and human Collagen Type I Rabbit 1:100 Novus Biologicals (NB600-408)
Mouse Collagen IV Rabbit 1:100 Novotec # 20451 0.5ml
Mouse Smooth Muscle Actin (ASM-1) Biotin 1:100 Progen # BK61501-1mg
Mouse LYVE-1 Rabbit 1:50 abcam # ab14917
Human CD31 Clone JC70A Mouse ready to use DAKO Code IR610
Human CD68 Clone PG-M1 Mouse ready to use DAKO Code GA613

Secondary antibodies
Antigen Conjugate Dilution Company & Catalogue number
Rat IgG (H+L) Alexa Fluor 488 1:350 Dianova # 712-546-153
Rat IgG (H+L) AMCA 1:50 Dianova # 712-155-153
Rabbit IgG (H+L) Alexa Fluor 488 1:500 Dianova # 711-546-152
Rabbit IgG (H+L) AMCA 1:50 Dianova # 111-155-003
Goat IgG (H+L) AMCA 1:50 Dianova # 705-155-147
Biotin Cy2 1:200 Dianova # 016-220-084

Validation

The antibodies were part of a routine pathological staining (for human samples) or validated on murine tumour sections, and have been routinely used. The antibodies were used as suggested by the supplier and have been used in several studies (e.g. Moss, Jennifer I., et al. "High-resolution 3D visualization of nanomedicine distribution in tumors." Theranostics 10.2 (2020): 880.; Theek, Benjamin, et al. "Histidine-rich glycoprotein-induced vascular normalization improves EPR-mediated drug targeting to and into tumors." Journal of controlled release 282 (2018): 25-34.; Doleschel, Dennis, et al. "Regorafenib enhances anti-PD1 immunotherapy efficacy in murine colorectal cancers and their combination prevents tumor regrowth." Journal of Experimental & Clinical Cancer Research 40.1 (2021): 1-14.)

Eukaryotic cell lines

Policy information about cell lines and Sex and Gender in Research

Cell line source(s)

A431 (SigmaAldrich; female human patient), MLS (ATCC; female human patient), CT26 (ATCC; female mouse), SW620 (ATCC; male human patient), A549 (ATCC; male human patient), Calu-3 (ATCC; male human patient), Calu-6 (ATCC; female human patient), 4T1 (ATCC; female mouse), Hep-55.1.C (CLS; female mouse).

Authentication

The cell lines were only inspected by eye.

Mycoplasma contamination

The cell lines regularly tested negative for mycoplasma contamination (via PCR).

Commonly misidentified lines
(See ICLAC register)

No commonly misidentified cell lines were used.

Animals and other research organisms

Policy information about studies involving animals; ARRIVE guidelines recommended for reporting animal research, and Sex and Gender in Research

Laboratory animals

Female CD1-nude, 6–8 weeks old. Female NMRI nude, 4–6 weeks old. Male CB.17 SCID, >18 g. Female CB.17 SCID, >18 g. Female Hsd:Athymic Nude-Foxn1nu, > 18 g. Female BALB/c mice, > 18 g. Male C57BL/6J mice, > 18 g.

Wild animals

The study did not involve wild animals.

Reporting on sex

Data were only reported in one sex per model but both sexes were used. Sex is not expected to impact the microenvironment of the tumour, so it was not deemed necessary to split established models across both sexes. We used male mice for the prostate cancer models, and female mice for the breast cancer models.

Field-collected samples

The study did not involve samples collected from the field.

Ethics oversight

All animal experiments were approved by the responsible governmental review committees on animal care. All work conducted in the UK adhered to the Animal Scientific Procedures Act 1986 and complied with the Global Bioethics Policy. All experiments were detailed in approved project licenses outlining the exact type of research performed, and initially went through internal ethical review processes, followed by assessment and approval by the LANUV (Germany) and Home Office (UK).

Note that full information on the approval of the study protocol must also be provided in the manuscript.

Clinical data

Policy information about clinical studies

All manuscripts should comply with the ICMJE guidelines for publication of clinical research and a completed CONSORT checklist must be included with all submissions.

Clinical trial registration

The trial was registered at the Medical Faculty of RWTH Aachen University (at the ethics committee, EK No. 22-294 and at the clinical trial center CTC-A, No. 21-359).

Study protocol

The study protocol was not published, as only a retrospective study on samples from the archive of the Institute of Pathology was performed.

Data collection

For the analysis of the tumour macrophages and blood vessels, samples (from tumour resections and biopsies) from breast (topography code: C50, ICD-O code: 8500/3 Infiltrating duct carcinoma, NOS), lung (topography code: C34, ICD-O code: 8070/3 squamous cell carcinoma, not other specified) and head and neck (topography codes: C02-C13, C32, C44, ICD-O code: 8070/3 squamous cell carcinoma, not other specified) were identified and retrieved from the routine diagnostic archive from the Institute of Pathology at RWTH Aachen University Hospital. In accordance with patient characteristics provided by Harrington et al. (Clin Cancer Res. 7, 243; 2001), tumours with a locally advanced (T3–T4) tumor stage according to the TNM classification were selected. Patients had not undergone neoadjuvant chemotherapy prior to surgery.

Tumour sections were stained with standard pathology protocols for CD31 and CD68.

Outcomes

Tumour blood vessels and macrophages were counted and correlated with the known liposome accumulation of breast, lung and head-and-neck cancer on the basis of accumulation values reported by Harrington et al. (Clin Cancer Res. 7, 243; 2001).
